# Serum N-Glycans: A New Diagnostic Biomarker for Light Chain Multiple Myeloma

**DOI:** 10.1371/journal.pone.0127022

**Published:** 2015-06-15

**Authors:** Jie Chen, Meng Fang, Yun-Peng Zhao, Chang-Hong Yi, Jun Ji, Cheng Cheng, Meng-Meng Wang, Xing Gu, Quan-Sheng Sun, Xiao-Ling Chen, Chun-Fang Gao

**Affiliations:** 1 Department of Laboratory Medicine, Eastern Hepatobiliary Surgery Hospital, Second Military Medical University, Shanghai, China; 2 Department of Hematology, Shanghai Zhabei District Central Hospital, Shanghai, China; National Cancer Institute at Frederick, UNITED STATES

## Abstract

The aim of this study was to evaluate the diagnostic and differential diagnostic power of serum N-glycans for light chain multiple myeloma (LCMM). A total of 167 cases of subjects, including 42 LCMM, 42 IgG myeloma, 41 IgA myeloma, and 42 healthy controls were recruited in this study. DNA sequencer-assisted fluorophore-assisted capillary electrophoresis (DSA-FACE) was applied to determine the quantitive abundance of serum N-glycans. The core fucosylated, bisecting and sialylated modifications were analyzed in serum of LCMM patients (n=20) and healthy controls (n=20) randomly selected from the same cohort by lectin blot. Moreover, serum sialic acid (SA) level was measured by enzymatic method. We found two N-glycan structures (NG1A2F, Peak3; NA2FB, Peak7) showed the optimum diagnostic efficacy with area under the ROC curve (AUC) 0.939 and 0.940 between LCMM and healthy control. The sensitivity and specificity of Peak3 were 88.1% and 92.9%, while Peak7 were 92.9% and 97.6%, respectively. The abundance of Peak3 could differentiate LCMM from IgG myeloma with AUC 0.899, sensitivity 100% and specificity 76.2%, and Peak7 could be used to differentiate LCMM from IgA myeloma with AUC 0.922, sensitivity 92.9% and specificity 82.9%. Serum SA level was significantly higher in patients with LCMM than that in healthy controls. Moreover, the decreased core fucosylation, bisecting and increased sialylation characters of serum glycoproteins were observed in patients with LCMM. We concluded that serum N-glycan could provide a simple, reliable and non-invasive biomarker for LCMM diagnosis and abnormal glycosylation might imply a new potential therapeutic target in LCMM.

## Introduction

Multiple myeloma (MM) is a kind of malignant disease characterized by abnormal proliferation of plasmacyte and monoclone immunoglobulin or free light chain (FLC). The latest statistics show that the annual incidence of MM is 7.74 per 100,000, mortality 3.52, mostly occurred in elder people and males [1]. The main paraprotein types of MM include IgG, IgA and light chain of immunoglobulins. Compared with IgG MM and IgA MM, an earlier average age of onset, more severe renal lesion and shorter overall survival appeared to occur in light chain multiple myeloma (LCMM) [2].

Serum protein electrophoresis (SPE), immunofixation electrophoresis (IFE) and serum FLC were recommended by international guideline to screen for MM and other plasma cell disorders [3–4]. However, the positive rates of SPE and IFE were not highly enough to identify LCMM [5]. As serum FLC derives from intact immunoglobulin or monoclonal light chain, the quantitative determination of serum FLC has been confirmed to be sensitive and specific in many monoclonal plasma cell diseases, such as all types of MM, monoclonal gammopathy of undetermined significance (MGUS) and amyloidosis [6–11], However, there is still lack of high sensitive and specific biomarker exclusive for LCMM.

As we all known, besides gene and protein expression, protein posttranslational modification (PPM) also reserves multiple biological functions to influence disease phenotype. Glycosylation is one of the most common and important mode in more than 200 PPMs [12–13]. Abnormal glycosylation is present in many autoimmune diseases [14–16] and various cancers [17–20]. The glycosylation traits on N-linked glycans are rarely revealed in LCMM. In this research, we introduce new noninvasive and convenient biomarker based on serum N-glycan abundance to diagnose and differentially diagnose LCMM.

## Methods

### Ethics Statement

The study protocol was approved by the Chinese Ethics Committee of Human Resources at the Second Military Medical University. Written informed consent was obtained from all study participants.

### Patients and serum samples

In all, 125 MM patients consisted of 42 LCMM, 42 IgG MM and 41 IgA MM together with 42 healthy controls were recruited from March 2010 to April 2012 in this study. MM patients were consecutive in-patients at Shanghai Zhabei District Central Hospital, and healthy donors were physical examination people at the same time. The healthy individual was defined as someone who was deemed free of diseases (including no history of cancer) at health examination. All MM patients were defined based on the International Myeloma Working Group (IMWG) criteria 2003 [Clonal bone marrow plasma cells ≥ 10%, presence of serum and/or urinary monoclonal protein (except in patients with true non-secretory multiple myeloma), and evidence of end-organ damage that can be attributed to the underlying plasma cell proliferative disorder] and staged according to ISS staging criteria after a confirmed diagnosis [21–22]. Both the patients newly diagnosis as MM and confirmed MM patients who received routine chemotherapy were included in the study. MM patients who had serious infectious diseases, acute or chronic inflammatory diseases, other malignant cancer history besides MM, and drug abuse were excluded. Hereinto, 14 LCMM, 12 IgG MM and 12 IgA MM were newly diagnosed without treatment, whereas 28 LCMM, 30 IgG MM and 29 IgA MM had been undergone the routine chemotherapy. Serum samples from newly diagnosed and treated patients were obtained before chemotherapy and before the next chemotherapy, respectively. Blood was collected using a standard protocol and serum samples were separated by centrifuging at 3000 rpm for 10 min, and then stored at −80°C.

### Laboratory tests and clinical information

The hematological indexs, hemoglobin (Hb) was determined by automatic cell counter and matched reagent (Sysmex XZ-2100D Cell Counter, Sysmex, Kobe, Japan; Sysmex diagnostic reagents). The biochemical indexs, such as total protein (TP), albumin (ALB), urea (BUN), and creatine (CREA) were measured by automatic chemical analyzer and matched reagent (ADVIA 2400 Analyzer, Siemens, Munich, Germany; Siemens diagnostic reagents). SPE and IFE were run by semi-auto electrophoresis system (Sebia HYDRASYS2 electrophoresis system, Sebia, Tours, France). Serum salic acid (SA) concentration (mg/dl) was detected using the enzymatic method automatically (Hitachi 7600 Analyzer, Hitachi, Tokyo, Japan; Wako diagnostic reagents, Wako Pure Chemical Industries Ltd., Osaka, Japan).

### Serum protein N-glycan profiling

Serum protein N-glycan analysis was performed using a DNA sequencer-assisted fluorophore-assisted capillary electrophoresis (DSA-FACE) technology as described previously [20]. Briefly, the N-glycans present on proteins in 2ul of serum were released with peptide N-glycosidase-F (PNGaseF) (New England Biolabs, Boston, Mass) and afterwards labeled with APTS (8-aminonaphtalene-1,3,6-trisulphonic acid) (Invitrogen, Carlsbad, Calif). Sialic acid was removed with arthrobacter ureafaciens sialidase (Roche Bioscience, Palo Alto, Calif) and the processed samples were analyzed using a capillary electrophoresis-based ABI3500 Genetic Analyzer (Applied Biosystems, Foster city, Calif). A total of 12 obvious N-glycan peaks detected in all serum samples were analyzed using the GeneMapper v4.1 software (Applied Biosystems). The abundance of each N-glycan peak was described by normalizing its height to the sum of the heights of all 12 peaks.

### Serum lectin blot

Serum samples from 20 LCMM patients and 20 healthy controls were randomly selected for lectin blot analysis. A total of 30 ug serum total proteins were separated by electrophoresis in 10% sodium dodecyl sulfate polyacrylamide gel (SDS-PAGE). Afterward, gels were stained with coomassie blue (CBB) G250 or the proteins separated in gels were transferred to a nitrocellulose membrane (Whatman/Schleicher&Schuell France, Versailles, France) for the detection of core-α-1,6-fucosylated, bisecting and sialylated glycoproteins. The membranes were blocked overnight at 4°Cwith 5% bovine serum albumin in Tris-buffered saline [140 mM NaCl, 10 mM Tris-HCl (TBS)] and then incubated for 2 hours at room temperature with 5 ug/mL of biotinylated Lens culinaris agglutinin (LCA), Phaseolus vulgaris erythroagglutinin (PHA-E), Maackia Amurensis Lectin II (MAL II) or Sambucus Nigra Agglutinin (SNA) (Vector Laboratories, Burlingame, Calif) in TBS containing 0.1% Tween-20 (TBST). After 4 washes for 10 minutes each with TBST, the membranes were incubated with a 1:10,000 dilution of IRDye 800CW-streptavidin (LI-COR Biosciences, Lincoln, Neb) for 1 hour at room temperature. After another 4 washes for 10 minutes each with TBST, the membranes were scanned using the Odyssey Infrared Imaging System (LI-COR Biosciences, Lincoln, Neb). The total protein stained with CBB was used to calculate the relative level of specific glycosylated proteins.

### Statistical analysis

All samples were analyzed in a randomized order and in a blinded manner, and the statistical analysis was independently performed by two researchers. All quantitative variables are expressed as means ± SD (standard deviations). Quantitative variables were compared with Student t test, ANOVA analysis or nonparametric test. Pearson coefficients of correlation and their associated probability (*P*) were used to evaluate the relationship between parameters. The diagnostic and differentially diagnostic performances of single marker were evaluated by the Receiver Operating Characteristic (ROC) curve analysis and the area under the curve (AUC). Sensitivity, specificity, positive predictive value (PPV), negative predictive value (NPV), and accuracy were calculated using cut-off value optimally selected upon the ROC curves. All reported *P* values were 2-tailed, and *P* values < 0.05 were evaluated as statistically significant. Statistical analyses were performed with SPSS16.0 for Windows software (SPSS, Chicago, IL).

## Results

### Characteristic change of N-glycan profiling patterns in LCMM

**Fig 1:**
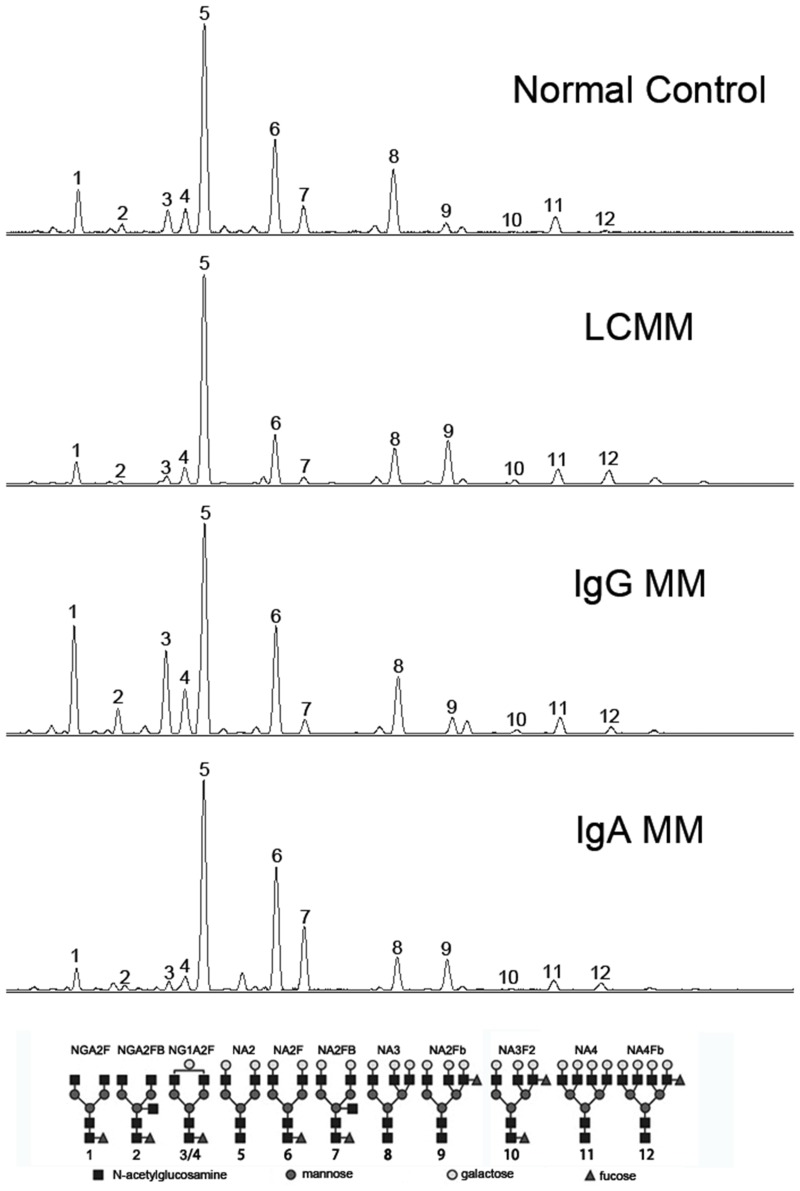
A representative N-glycan profiling of total serum glycoproteins. At least 12 peaks can be identified by DNA sequencer-assisted fluorophore-assisted capillary electrophoresis (DSA-FACE) technology in four groups. The structure of the N-glycan peaks and the symbols indicated the corresponding glycans are displayed below the panel.

**Fig 2:**
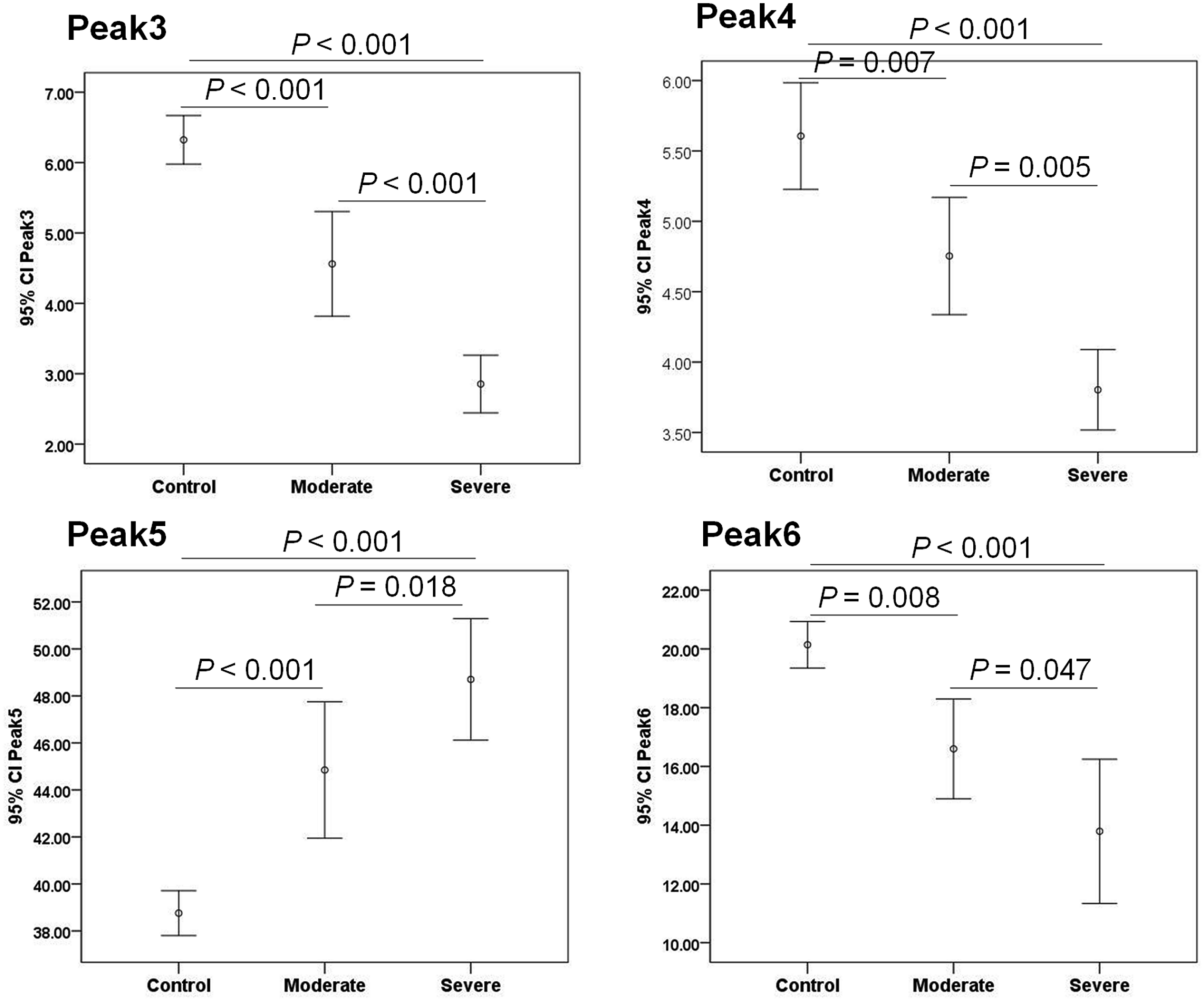
Peak abundances for the N-glycans which had the obvious overall changes among control subjects, moderate and severe LCMM subjects. Peak3, Peak4 and Peak6 decreased, whereas Peak5 increased associated with the severity of LCMM disease. Error bars are 95% confidence interval (95% CI) of the means.

The basic clinical and laboratory data were summarized in Table 1. As shown in Fig 1, at least 12 obvious N-glycan peaks were identified in all serum samples. The structure of these peaks were determined previously by Callewaert et al and Liu et al [18,23]. The abundance difference of N-glycan peaks in 4 different groups was shown in Table 2. Most of the biantennary structure levels (Peak2, 3, 4, 6 and 7) were decreased remarkably in LCMM compared with those in healthy control, whereas a biantennary glycan (Peak 5), the triantennary glycans (Peak8, 9 and 10) and the tetra-antennary glycans (Peak11 and 12) increased significantly. When compared to IgG MM and IgA MM, the abundance of peak5, 8, 9, 10, 11 and 12 were also increased in LCMM patients. Two biantennary glycans (NA2F, Peak6; NA2FB, Peak7) were the lowest in LCMM among all groups, and Peak7 was prominently increased just in IgA patients. Additionally, two monogalactosyl N-glycans (NG1A2F, Peak3 and Peak4) were decreased in LCMM than those in IgG MM but increased than those in IgA MM, while Peak3 showed extremely higher only in IgG patients. Moreover, we combined the ISS stage I with the ISS stage II as the moderate group (n = 14), granted the ISS stage III as the severe group (n = 28), and found that the abundance of Peak3, 4, 5 and Peak6 were significantly changed associated with the severity of the disease, indicating that these glycans could potentially be used as markers to track the progression of LCMM (Fig 2). Additionally, most N-glycan peaks showed no significant differences between MM patients with or without treatment (S1 Table), and between MM patients with kappa or lambda light chain M proteins (S2 Table).

**Table 1:** The laboratory tests and basic clinical information in three MM groups and healthy control group.

M protein type	Healthy control (n = 42)	LCMM(κ 19, λ 23)	IgG MM(κ 20, λ 22)	IgA MM (κ 22, λ 19)
Male [n(%)]	25 (59.5%)	26 (61.9%)	25 (59.5%)	23 (56.1%)
Age (year)	59.14 ± 5.90	58.48 ± 12.15	64.81 ± 11.87	67.83 ± 11.10
Hemoglobin (g/L)	146.34 ± 9.42	99.37 ± 28.70	107.07 ± 27.28	98.78 ± 28.85
ISS stage I [n(%)]	/	11 (26.2%)	11 (26.2%)	12 (29.3%)
ISS stage II [n(%)]	/	3 (7.1%)	16 (38.1%)	16 (39.0%)
ISS stage III [n(%)]	/	28 (66.7%)	15 (35.7%)	13 (31.7%)
Platelet (×10^9^/L)	202.68 ± 51.21	168.34 ± 84.11	154.21 ± 82.54	162.83 ± 124.58
Total protein (g/L)	76.24 ± 3.87	61.27 ± 8.19	72.75 ± 18.46	73.02 ± 16.01
Albumin (g/L)	47.17 ± 2.18	39.17 ± 5.46	35.50 ± 8.10	32.47 ± 7.58
Urea (mmol/L)	5.84 ± 1.04	10.30 ± 6.58	5.62 ± 2.05	6.12 ± 3.29
Creatinine (μmol/L)	72.40 ± 10.87	198.20 ± 215.19	67.44 ± 18.13	85.65 ± 71.58
SPE positive [n(%)]	/	12 (28.6%)	40 (95.2%)	24 (58.5%)
IFE positive [n(%)]	/	24 (57.1%)	41(97.6%)	36 (87.8%)

Note: Measurement data are expressed as means ± standard deviations; enumeration data are expressed as n (%); / represents no related data.

Abbreviations: ISS, International Staging System; SPE, serum protein electrophoresis; IFE, immunofixation electrophoresis.

**Table 2:** Abundance of N-glycan profilings in LCMM, IgG MM, IgA MM patients and healthy controls.

Peaks	Healthy control (n = 42)	LCMM (κ 19, λ 23)	IgG MM (κ 20, λ 22)	IgA MM (κ 22, λ 19)	*P1*	*P2*	*P3*
Peak 1	7.79 ± 1.94	6.95 ± 2.96	13.68 ± 12.48	4.82 ± 3.25	NS	0.001	0.001
Peak 2	1.30 ± 0.45	0.91 ± 0.54	1.52 ± 2.50	0.65 ± 0.41	<0.001	NS	0.022
Peak 3	6.32 ± 1.11	3.42 ± 1.39	11.97 ± 8.38	2.50 ± 1.85	<0.001	<0.001	0.003
Peak4	5.61 ± 1.22	4.12 ± 0.85	6.31 ± 2.54	3.24 ± 1.02	<0.001	<0.001	<0.001
Peak 5	38.76 ± 3.06	47.42 ± 6.37	30.82 ± 13.86	40.59 ± 8.86	<0.001	<0.001	<0.001
Peak 6	20.14 ± 2.54	14.73 ± 5.56	21.16 ± 10.45	22.45 ± 7.77	<0.001	<0.001	<0.001
Peak 7	6.21 ± 1.40	2.68 ± 2.61	3.33 ± 3.46	13.28 ± 12.93	<0.001	NS	<0.001
Peak 8	7.80 ± 1.96	10.03 ± 3.48	5.90 ± 2.96	6.15 ± 2.92	0.002	<0.001	<0.001
Peak 9	2.68 ± 1.28	5.04 ± 3.18	2.80 ± 1.89	3.46 ± 2.27	<0.001	<0.001	0.017
Peak 10	0.34 ± 0.13	0.46 ± 0.23	0.31 ± 0.23	0.36 ± 0.22	0.039	<0.001	0.043
Peak 11	1.69 ± 0.61	3.01 ± 1.34	1.57 ± 0.82	1.66 ± 0.85	<0.001	<0.001	<0.001
Peak 12	0.47 ± 0.24	1.23 ± 0.72	0.64 ± 0.44	0.84 ± 0.60	<0.001	<0.001	0.009

Note: Measurement data are expressed as means ± standard deviations;

*P1*: comparison between LCMM and healthy control,

*P2*: comparison between LCMM and IgG MM,

*P3*: comparison between LCMM and IgA MM;

Abbreviations: LCMM, light chain multiple myeloma;

IgG MM, IgG type multiple myeloma;

IgA MM, IgA type multiple myeloma;

NS, none-significant.

### N-glycan markers were correlated well with clinical parameters about end-organ damage

Several clinical parameters had been chosen to access the basic condition of the patients. Hb is usually applied to evaluate the anemia degree. BUN and CREA can reflect the renal malfunction. TP and ALB represent protein non-homeostasis in patients. The correlation between 12 N-glycan markers and clinical parameters was listed in Table 3. The significantly decreased peaks (Peak 3, 4, 6 and 7) had positive correlation with Hb, TP and ALB, accompanied by negative correlation with BUN and CREA. The significantly increased peaks (Peak 5, 8, 9, 10, 11 and 12) were positively correlated to BUN and CREA, and negatively correlated to Hb, TP and ALB.

**Table 3:** Correlation between N-glycan markers and clinical indexes in LCMM patients and healthy controls.

Correlations		Peak1	Peak2	Peak3	Peak4	Peak5	Peak6	Peak7	Peak8	Peak9	Peak10	Peak11	Peak12
Hb	*r*	0.338	0.453	0.727	0.581	-0.507	0.405	0.390	-0.328	-0.478	-0.333	-0.495	-0.621
	*P*	0.002	<0.001	<0.001	<0.001	<0.001	<0.001	<0.001	0.003	<0.001	0.002	<0.001	<0.001
TP	*r*	0.279	0.360	0.690	0.467	-0.615	0.543	0.655	-0.380	-0.517	-0.413	-0.528	-0.610
	*P*	0.010	0.001	<0.001	<0.001	<0.001	<0.001	<0.001	<0.001	<0.001	<0.001	<0.001	<0.001
ALB	*r*	0.266	0.398	0.643	0.434	-0.475	0.475	0.471	-0.316	-0.571	-0.536	-0.433	-0.664
	*P*	0.014	<0.001	<0.001	<0.001	<0.001	<0.001	<0.001	0.003	<0.001	<0.001	<0.001	<0.001
BUN	*r*	-0.039	-0.206	-0.429	-0.337	0.430	-0.409	-0.343	0.024	0.440	0.288	0.147	0.484
	*P*	NS	NS	<0.001	0.002	<0.001	<0.001	0.002	NS	<0.001	0.008	NS	<0.001
CREA	*r*	-0.093	-0.151	-0.420	-0.311	0.417	-0.327	-0.297	-0.103	0.501	0.334	-0.012	0.455
	*P*	NS	NS	<0.001	0.004	<0.001	0.003	0.006	NS	<0.001	0.002	NS	<0.001

Note: Abbreviations: Hb: hemoglobulin; TP: total protein; ALB: albumin; BUN: blood urea nitrogen; CREA: creatinine.

### Assessment of the N-glycan biomarkers for diagnosis and differentially diagnosis of LCMM

**Fig 3:**
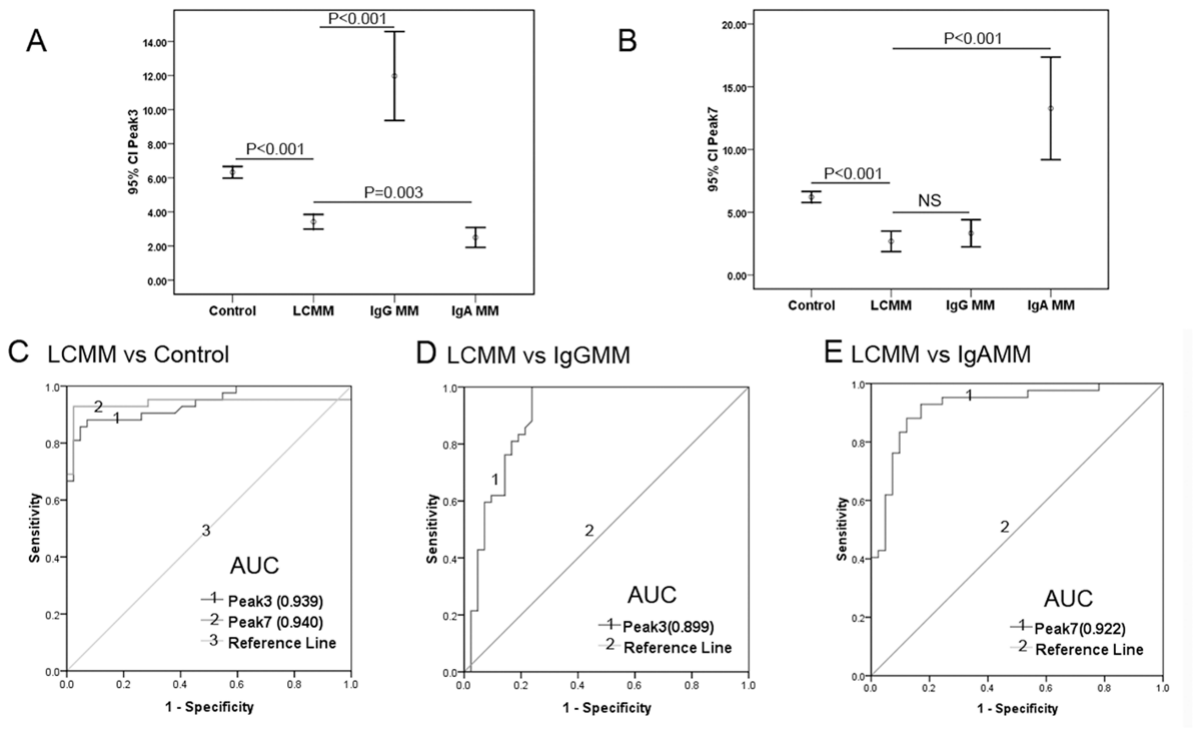
Peak3 and Peak7 could be used as diagnosis and differential diagnosis markers for LCMM. (A and B): showed the changes of two biantennary glycans (NG1A2F, Peak 3; and NA2FB, Peak 7) among 4 groups and error bars represent 95% confidence intervals (95% CI) of the means. Peak3 and Peak7 were decreased in LCMM group compared with the other 3 groups, except that Peak 3 was elevated in patients with LCMM relative to patients with IgA MM. (C): ROC curves for Peak 3 and Peak7 in patients with LCMM versus healthy controls with the area under the curve (AUC) of 0.939 and 0.940, respectively. (D): ROC curve for Peak3 in patients with LCMM versus IgG MM and the AUC was 0.899. (E): ROC curve for Peak7 in patients with LCMM versus IgA MM with the AUC of 0.922.

The diagnostic powers of N-glycans for defining LCMM referred to healthy control, and differentiating LCMM from IgG MM or IgA MM were calculated by ROC curve and AUC analysis. Peak3 as well as Peak7 revealed the best diagnostic powers among all 12 N-glycan peaks to distinguish LCMM from healthy individuals with the AUC of 0.939 and 0.940, respectively. And the optimum diagnostic cut-off values for Peak3 and Peak 7 were 5.205 (sensitivity 88.1%, specificity 92.9%, accuracy 90.5%) and 4.105 (sensitivity 92.9%, specificity 97.6%, accuracy 95.2%), respectively (Fig 3C, Table 4). More interestingly, when compared to healthy controls, both Peak3 and Peak7 were lower in LCMM subjects, whereas in IgG MM patients, Peak7 was lower but Peak3 was dramatically higher, and conversely, Peak3 was lower but Peak7 was predominantly increased in IgA MM patients (Fig 3A and 3B). Peak3 showed the best diagnostic power to differentiate LCMM from IgG MM patients (AUC = 0.899) with the optimum cut-off value as 6.855 (sensitivity 100.0%, specificity 76.2%, accuracy 88.1%), while Peak7 showed the greatest AUC to distinguish LCMM from IgA MM patients (AUC = 0.922) with the optimum cut-off value as 4.220 (sensitivity 92.9%, specificity 82.9%, accuracy 88.0%) (Fig 3D and 3E, Table 4).

**Table 4:** Sensitivity and specificity of N-glycan biomarkers for the diagnosis and differential diagnosis of LCMM.

Cut off value	Sensitivity (%)	Specificity (%)	PPV (%)	NPV (%)	Accuracy (%)
Peak3 (LCMM vs Healthy control) (5.205)	88.1	92.9	92.5	88.6	90.5
Peak7 (LCMM vs Healthy control) (4.105)	92.9	97.6	97.5	93.2	95.2
Peak3 (LCMM vs IgG MM) (6.855)	100.0	76.2	80.8	100.0	88.1
Peak7 (LCMM vs IgA MM) (4.220)	92.9	82.9	84.8	91.9	88.0

Note: Abbreviations: PPV: positive predictive value; NPV: negative predictive value.

### Decreased levels of core-fucolysation and bisecting proteins in LCMM

**Fig 4:**
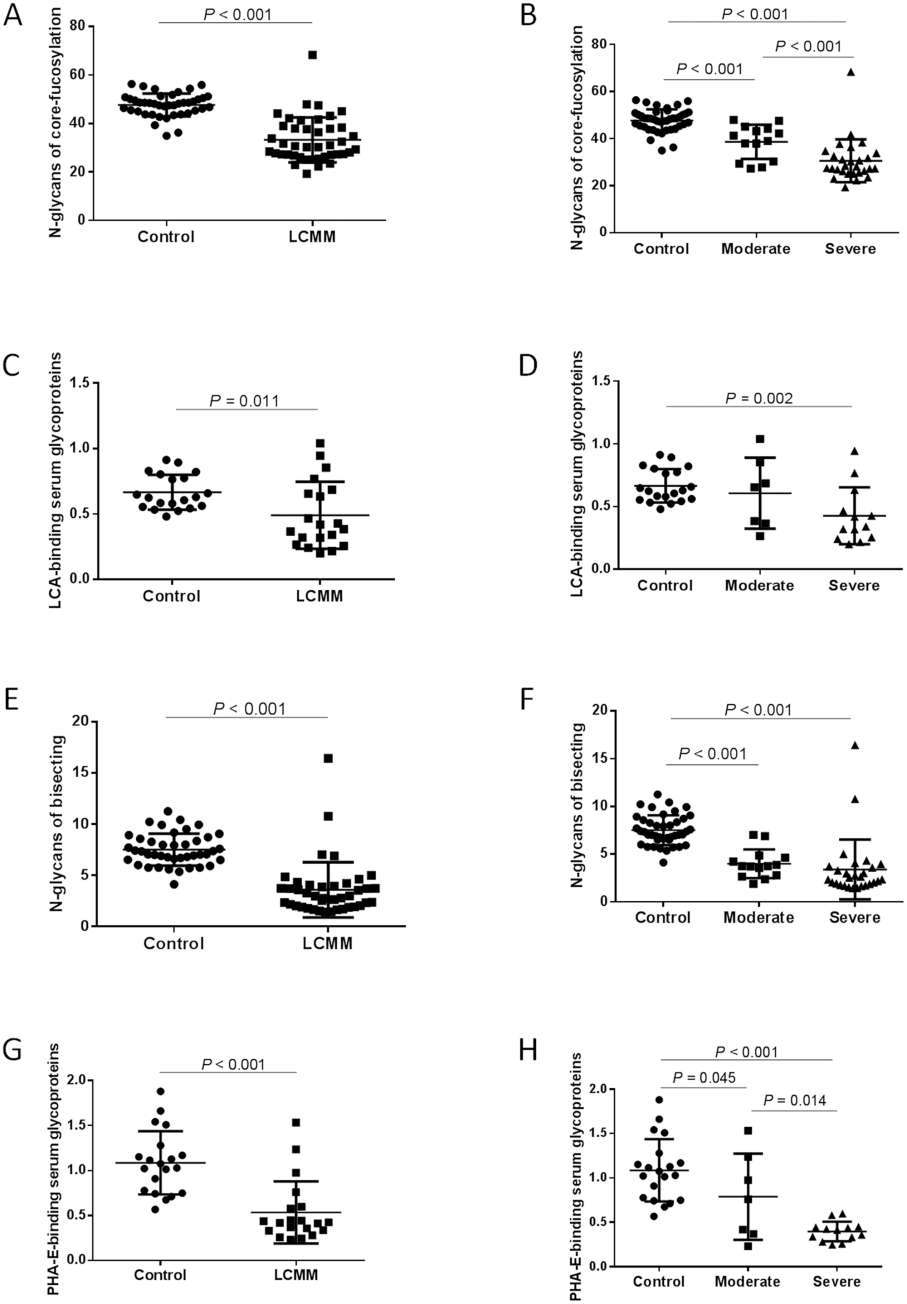
The abundance of core fucosylation and bisecting modification in N-glycan and glycoprotein levels. The abundance of core fucosylation and bisecting modification in N-glycan and glycoprotein levels were illustrated using DSA-FACE and serum lectin blot. (A) and (B): The abundance of total core fucosylation residues (the sum of Peak1, 2, 3, 4, 6, 7 and Peak10) from DSA-FACE decreased in LCMM patients (n = 42) than that in healthy controls (n = 42), and decreased according to the severity of the disease [moderate LCMM (n = 14) and severe LCMM (n = 28)]. (C) and (D): LCA binding core-fucosylated glycoprotein level detected by serum lectin blot was decreased in LCMM patients (n = 20) than that in healthy controls (n = 20). The level of serum core-fucosylated glycoprotein was significantly lower in severe LCMM (n = 13) but not in moderate LCMM (n = 7). The vertical axis indicates the ratio of fluorescence intensity of fucosylated proteins to total proteins stained by coomassie blue (CBB). (E) and (F): The abundance of total bisecting residues (the sum of Peak2 and Peak7) from DSA-FACE deceased in LCMM patients than that in healthy controls, and decreased associated with the severity of disease. (G) and (H): PHA-E binding bisecting glycoprotein level detected by lectin blot was decreased in LCMM patients and decreased associated with the severity of disease. The abundance on vertical axis was represented as mean ± standard deviation.

The total core fucose (the sum of Peak1, 2, 3, 4, 6, 7, Peak10) and bisecting β-1,4-N-acetyl glucosamine (the sum of Peak2 and Peak7) residues of serum N-glycoproteins were significantly lower (*P* < 0.001; *P* < 0.001, resp.) in LCMM than those in healthy controls (Fig 4A and 4E). The decreased level of core fucosylated and bisecting N-glycans were associated with the severity of LCMM (Fig 4B and 4F). In order to validate the glycosylation pattern in serum glycoproteins, lectin bolt was performed in serum from 20 healthy controls and 20 LCMM patients including 7 cases of moderate stage and 13 cases of severe stage. We probed the core fucosylated and bisecting glycoproteins using lectins LCA and PHA-E, since LCA can specifically recognize the glycoproteins with core α-1,6-fucosylated-linked N-acetyl-D-glucosamine-asparagine (GlcNAc-Asp) in the trimannosyl core, and PHA-E can specifically binding to bisecting β-1,4-linked N-acetylglucosamine. The serum levels of both LCA-binding core fucosylated glycoproteins and PHA-E-binding bisecting glycoproteins were lower in LCMM group than those in control group (*P* = 0.011 and *P* < 0.001, resp.), which supported the finding in serum DSA-FACE (Fig 4C and 4G). The abundance of LCA-binding proteins in severe group was significantly lower than that in control group (*P* = 0.002) (Fig 4D). Moreover, the abundance of PHA-E-binding proteins in moderate and severe groups were obviously decreased than those in control group (*P* = 0.045 and *P* < 0.001 resp.) (Fig 4H).

### Increased levels of sialylation proteins and serum SA in LCMM

**Fig 5:**
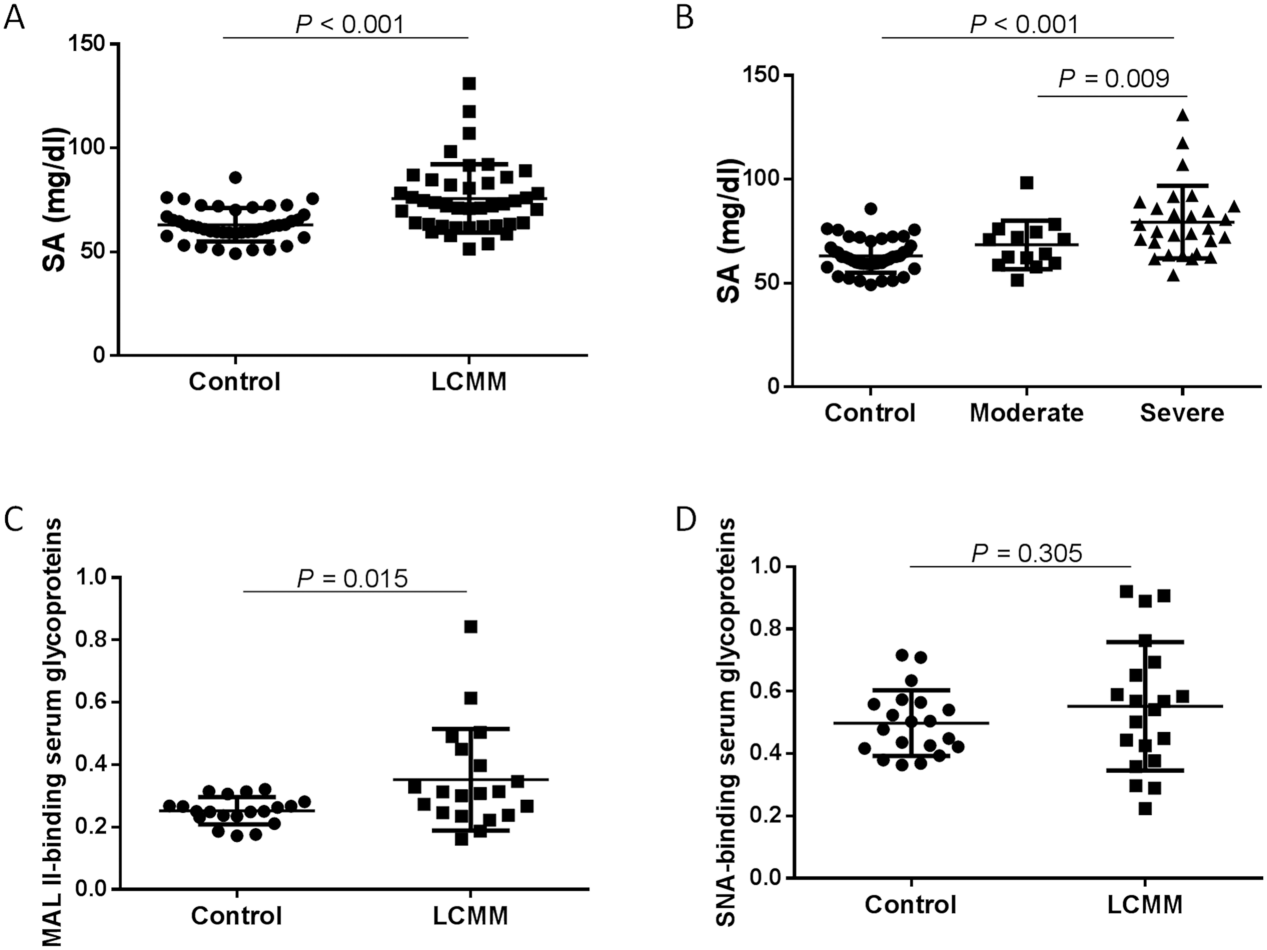
The levels of serum salic acid (SA) and sialylated glycoproteins. The increased levels of serum salic acid (SA) and sialylated glycoproteins in LCMM patients were clarified by enzymatic method and using lectin blot, respectively. (A): The horizontal axis represents the experimental groups: control (n = 42), LCMM (n = 42). The vertical axis indicates the serum SA level (mg/dl), and error bars are mean ± standard deviations. (B) and (C): Lectin blots of serum glycoproteins were probed with MAL II and SNA representing the α-2,3-linked and α-2,6-linked sialylated proteins. The horizontal axis represents the experimental groups: control (n = 20) and LCMM (n = 20). The vertical axis indicates the fluorescence intensity ratio of MAL II or SNA binding proteins to total proteins stained by coomassie blue (CBB), and error bars are mean ± standard deviations. MAL II binding α-2,3-linked sialylated protein increased in LCMM patients, whereas there was no significant change in SNA binding α-2,6-linked sialylated proteins between LCMM patients and controls.

Due to DSA-FACE only examining the N-glycan profiling in desialyated form, we took great interest in the sialylation modification state in LCMM. There was significant increase in the serum SA concentration (mg/dl) of patients with LCMM when compared to healthy controls (*P* < 0.001). Further analysis revealed that the SA level in severe LCMM group was significantly higher than that in healthy control group and moderate LCMM group (*P* < 0.001; *P* = 0.009, resp.) (Fig 5A and 5B). Two kinds of salic acid specific binding lectins MAL II and SNA were used to investigate the α-2,3-linked and α-2,6-linked sialylated proteins in serum by lectin blot technology. We found the serum level of MAL II binding α-2,3-linked sialylated protein was higher in the LCMM group than that in the control group (*P* = 0.015), but there was no significant difference of SNA binding α-2,6-linked sialylated protein between two groups (Fig 5C and 5D).

## Discussion

The majority of serum proteins are glycosylated and their glycan parts have numerous structural, functional and regulatory roles [24]. Changes in the glycosylation patterns of proteins play a pivotal role in many physiological processes, including protein folding and rearrangement, cell-cell and cell-matrix interaction, cell differentiation, proliferation and immune response [25–26]. DSA-FACE, which we applied in this study, provides a convenient, non-invasive, excellent sensitive method to detect desialylated N-glycan profiling in serum and may be more practical for routine clinical analysis. This technique was previously used to assist in the diagnosis of various cancers, such as Hepatocellular Carcinoma (HCC), colorectal cancer and gastric cancer [19,20,27]. As we have mentioned before, SPE and IFE are poor at detecting free light chains. Our study showed that the positive rates of SPE and IFE in LCMM subjects were 28.6% and 57.1%, respectively. A similar observation (36.4% and 61.5%) was previously reported by Chen NF et al [28]. Dimers or polymers of immunoglobulin light chains (usually λ dimers) interfere the determination of serum or urine FLC analysis [29–30]. Shaheen SP et al reported serum FLC analysis might miss monoclonal light chains that urine IFE detected [31]. Moreover, the sensitivity of urine FLC detection was restricted by the kidney capacity for reabsorbing and catabolizing FLC [6]. On the basis of statistical analysis according to serum DSA-FACE results, we found 2 N-glycan structures (NG1A2F, Peak3; NA2FB, Peak7) possessed excellent diagnostic efficacy (AUC = 0.939, and AUC = 0.940). More interestingly, compared with healthy controls, both Peak3 and Peak7 decreased in LCMM, whereas Peak3 increased but Peak7 deceased in IgG MM, conversely Peak3 decreased but Peak7 increased in IgA MM. In addition, Peak3 and Peak7 could significantly differentiate the patients with LCMM from those with IgG MM (AUC = 0.899) and IgA MM (AUC = 0.922), respectively. Meanwhile, Peak3 and Peak7 showed no significant differences between MM patients with or without treatment, and between MM with different type of light chain, which expounded that Peak3 and Peak7 were common markers for MM presenting disease-specificity. Thus we deduce that N-glycans Peak3 and Peak7 could be used as serum biomarkers for LCMM. Despite all this, a large scaled study will be necessary to confirm and validate these preliminary findings for the N-glycan biomarkers in the value of diagnosis and differential diagnosis between LCMM and other more plasma cell disorders, including Waldenström’s macroglobulinemia, MGUS, solitary plasmacytoma, systemic AL amyloidosis and POEMS syndrome. Additionally, the reference intervals for Peak3 and Peak7 in healthy individuals need to be defined in the further study with a larger sample size. Moreover, 4 of 12 N-glycan peaks had good correlations to disease severity which indicated them as monitoring indexes, and the N-glycan changes were in accordance with the fluctuation of clinical parameters which reflect the end-organ damage (anemia and renal damage).

Alterations in glycosylation are associated with numerous diseases and glycans are attracting increasing attention both as disease biomarkers and targets for novel therapeutic approaches. In this study, we observed the decreased core fucosylation and bisecting of serum N-glycan modification in LCMM subjects by using both DSA-FACE and lectin blot. Meanwhile, core fucosylated and bisecting features of N-glycoproteins adequately reflected the severity of LCMM. Moreover, increased sialylation (mainly derived from α-2,3-linked sialic acid) of serum proteins was confirmed by MAL II-binding lectin blot analysis in LCMM patients. The decreased core-fucosylation has also been reported in colorectal cancer, gastric cancer and breast carcinoma, and an increase in sialylation was also found in breast cancer [19, 32–33]. Recently, Glavey SV et al reported that high expression of ST3GAL6, an α-2,3-linked sialyltransferase, could influence homing and survival in multiple myeloma [34]. This suggests that the sialylation of proteins plays a significant role in the course of LCMM.

In conclusion, our work here showed that serum N-glycans would have the potential to be used as simple and robust biomarkers for routine diagnosis, differential diagnosis and monitoring of LCMM. Moreover, increased serum level of sialyaltion and decreased core fucosylation and bisecting which imply the alterations in the activity of glycosyltransferases and glycosidases would provide a novel therapeutic target to this incurable disease in future.

## Supporting Information

S1 Table Abundance of N-glycans between untreated and treated MM patients. (DOC)

S2 Table Abundance of N-glycans in MM patients with different type of light chain (κ and λ). (DOC)

S3 Table Full data of basic clinical information and N-glycan abundance using DSA-FACE. (DOC)
