# Supplementary material for: Serum N-Glycans: A New Diagnostic Biomarker for Light Chain Multiple Myeloma
Source: PLoS One. 2015 Jun 15;10(6):e0127022. doi: 10.1371/journal.pone.0127022 (PMC4468189; doi:10.1371/journal.pone.0127022)
Supplement: S1 Table — (DOC) [file pone.0127022.s001.doc]

**S1 Table** Abundance of N-glycans between untreated and treated MM patients

| Peaks | Healthy control | LCMM | | IgG MM | | IgA MM | | *P1* | *P2* | *P3* |
| --- | --- | --- | --- | --- | --- | --- | --- | --- | --- | --- |
|  | (n =42) | untreated  (n=14) | treated  (n=28) | untreated  (n=12) | treated  (n=30) | untreated  (n=12) | treated  (n=29) |  |  |  |
| Peak 1 | 7.79 ± 1.94 | 7.38 + 3.37 | 6.74 + 2.78 | 12.58 + 12.70 | 14.12 + 12.58 | 4.31 + 3.21 | 5.03 + 3.30 | NS | NS | NS |
| Peak 2 | 1.30 ± 0.45 | 1.21 + 0.73 | 0.76 + 0.34 | 1.04 + 1.10 | 1.71 + 2.87 | 0.58 + 0.40 | 0.68 + 0.41 | 0.045 | NS | NS |
| Peak 3 | 6.32 ± 1.11 | 3.26 + 1.22 | 3.51 + 1.48 | 14.26 + 8.79 | 11.06 + 8.18 | 2.06 + 1.71 | 2.68 + 1.90 | NS | NS | NS |
| Peak4 | 5.61 ± 1.22 | 3.98 + 1.00 | 4.19 + 0.78 | 6.39 + 2.11 | 6.27 + 2.72 | 2.99 + 1.04 | 3.35 + 1.01 | NS | NS | NS |
| Peak 5 | 38.76 ± 3.06 | 46.35 + 8.38 | 47.95 + 5.19 | 26.78 + 12.98 | 32.43 + 14.08 | 40.19 + 9.92 | 40.76 + 8.57 | NS | NS | NS |
| Peak 6 | 20.14 ± 2.54 | 15.04 + 8.66 | 14.57 + 3.28 | 26.62 + 13.01 | 18.97 + 8.54 | 25.90 + 8.73 | 21.03 + 7.00 | NS | 0.030 | NS |
| Peak 7 | 6.21 ± 1.40 | 3.87 + 4.10 | 2.09 + 1.05 | 2.55 + 2.50 | 3.64 + 3.77 | 13.27 + 14.05 | 13.28 + 12.71 | NS | NS | NS |
| Peak 8 | 7.80 ± 1.96 | 9.72 + 3.08 | 10.18 + 3.71 | 4.90 + 2.96 | 6.30 + 2.91 | 5.63 + 3.26 | 6.37 + 2.80 | NS | NS | NS |
| Peak 9 | 2.68 ± 1.28 | 4.76 + 2.70 | 5.18 + 3.43 | 2.64 + 1.45 | 2.87 + 2.05 | 2.62 + 2.81 | 3.81 + 1.95 | NS | NS | NS |
| Peak 10 | 0.34 ± 0.13 | 0.41 + 0.18 | 0.48 + 0.24 | 0.35 + 0.25 | 0.29 + 0.23 | 0.29 + 0.15 | 0.39 + 0.23 | NS | NS | NS |
| Peak 11 | 1.69 ± 0.61 | 2.82 + 0.78 | 3.10 + 1.56 | 1.29 + 0.79 | 1.68 + 0.82 | 1.53 + 0.90 | 1.71 + 0.84 | NS | NS | NS |
| Peak 12 | 0.47 ± 0.24 | 1.20 + 0.55 | 1.25 + 0.80 | 0.59 + 0.30 | 0.66 + 0.49 | 0.65 + 0.63 | 0.92 + 0.58 | NS | NS | NS |

Note: Measurement data are expressed as means + standard deviations; *P1*: comparison between untreated and treated LCMM, *P2*: comparison between untreated and treated IgG MM, *P3*: comparison between untreated and treated IgA MM.

Abbreviations: LCMM, light chain multiple myeloma; IgG MM, IgG type multiple myeloma; IgA MM, IgA type multiple myeloma; NS, none-significant
