# Supplementary material for: Serum N-Glycans: A New Diagnostic Biomarker for Light Chain Multiple Myeloma
Source: PLoS One. 2015 Jun 15;10(6):e0127022. doi: 10.1371/journal.pone.0127022 (PMC4468189; doi:10.1371/journal.pone.0127022)
Supplement: S2 Table — (DOC) [file pone.0127022.s002.doc]

**S2 Table** Abundance of N-glycans in MM patients with different type of light chain (κ and λ)

| Peaks | Healthy control | LCMM | | IgG MM | | IgA MM | | *P1* | *P2* | *P3* |
| --- | --- | --- | --- | --- | --- | --- | --- | --- | --- | --- |
|  | (n =42) | κ (n=19) | λ (n=23) | κ (n=20) | λ (n=22) | κ (n=22) | λ (n=19) |  |  |  |
| Peak 1 | 7.79 ± 1.94 | 7.34 + 2.75 | 6.64 + 3.16 | 13.14 + 10.30 | 14.17 + 14.41 | 4.72 + 3.37 | 4.93 + 3.19 | NS | NS | NS |
| Peak 2 | 1.30 ± 0.45 | 0.93 + 0.42 | 0.89 + 0.63 | 1.24 + 0.86 | 1.77 + 3.38 | 0.66 + 0.36 | 0.64 + 0.46 | NS | NS | NS |
| Peak 3 | 6.32 ± 1.11 | 3.55 + 1.48 | 3.31 + 1.33 | 11.19 + 8.14 | 12.69 + 8.72 | 2.27 + 1.64 | 2.77 + 2.08 | NS | NS | NS |
| Peak4 | 5.61 ± 1.22 | 4.30 + 0.74 | 3.97 + 0.93 | 6.12 + 1.98 | 6.48 + 2.99 | 3.19 + 1.06 | 3.30 + 1.00 | NS | NS | NS |
| Peak 5 | 38.76 ± 3.06 | 47.74 + 5.37 | 47.15 + 7.21 | 33.16 + 12.86 | 28.68 + 14.67 | 38.41 + 9.03 | 43.12 + 8.18 | NS | NS | NS |
| Peak 6 | 20.14 ± 2.54 | 14.39 + 2.95 | 15.00 + 7.09 | 20.09 + 7.43 | 22.13 + 12.70 | 22.89 + 9.04 | 21.94 + 6.19 | NS | NS | NS |
| Peak 7 | 6.21 ± 1.40 | 2.16 + 1.11 | 3.11 + 3.35 | 3.80 + 3.64 | 2.90 + 3.32 | 16.51 + 14.20 | 9.53 + 10.44 | NS | NS | NS |
| Peak 8 | 7.80 ± 1.96 | 10.22 + 3.77 | 9.87 + 3.29 | 6.53 + 3.08 | 5.33 + 2.79 | 5.55 + 2.36 | 6.84 + 3.39 | NS | NS | NS |
| Peak 9 | 2.68 ± 1.28 | 4.68 + 3.80 | 5.33 + 2.60 | 2.19 + 1.31 | 3.36 + 2.17 | 3.18 + 2.15 | 3.79 + 2.41 | NS | 0.038 | NS |
| Peak 10 | 0.34 ± 0.13 | 0.42 + 0.20 | 0.49 + 0.24 | 0.27 + 0.17 | 0.34 + 0.28 | 0.38 + 0.27 | 0.34 + 0.13 | NS | NS | NS |
| Peak 11 | 1.69 ± 0.61 | 3.12 + 1.61 | 2.92 + 1.11 | 1.75 + 0.84 | 1.41 + 0.78 | 1.43 + 0.58 | 1.92 + 1.04 | NS | NS | NS |
| Peak 12 | 0.47 ± 0.24 | 1.15 + 0.86 | 1.30 + 0.60 | 0.52 + 0.28 | 0.75+ 0.54 | 0.80 + 0.62 | 0.89 + 0.59 | NS | NS | NS |

Note: Measurement data are expressed as means + standard deviations; *P1*: comparison between κ and λ LCMM, *P2*: comparison between κ and λ IgG MM,
*P3*: comparison between κ and λ IgA MM.

Abbreviations: LCMM, light chain multiple myeloma; IgG MM, IgG type multiple myeloma; IgA MM, IgA type multiple myeloma; NS, none-significant
