# Supplementary material for: Serum N-Glycans: A New Diagnostic Biomarker for Light Chain Multiple Myeloma
Source: PLoS One. 2015 Jun 15;10(6):e0127022. doi: 10.1371/journal.pone.0127022 (PMC4468189; doi:10.1371/journal.pone.0127022)
Supplement: S3 Table — (DOC) [file pone.0127022.s003.doc]

**S3 Table** Full data of basic clinical information and N-glycan abundance using DSA-FACE

| No. | group^a^ | age | gender^b^ | LC^c^ | ISS stage | chemo^d^ | SPE^e^ | IFE^e^ | Hb | PLT | TP | ALB | BUN | CREA | SA | P1 | P2 | P3 | P4 | P5 | P6 | P7 | P8 | P9 | P10 | P11 | P12 |
| --- | --- | --- | --- | --- | --- | --- | --- | --- | --- | --- | --- | --- | --- | --- | --- | --- | --- | --- | --- | --- | --- | --- | --- | --- | --- | --- | --- |
| 1 | HC | 54 | M |  |  |  |  |  | 164 | 218 | 77 | 46 | 4.9 | 76 | 62.5 | 6.71 | 1.16 | 6.73 | 5.07 | 39.35 | 18.60 | 6.84 | 8.00 | 3.88 | 0.28 | 1.97 | 0.71 |
| 2 | HC | 54 | M |  |  |  |  |  | 146 | 157 | 77 | 49 | 5.2 | 68 | 57.7 | 5.64 | 0.79 | 5.21 | 4.56 | 42.24 | 21.52 | 5.73 | 8.64 | 2.10 | 0.24 | 2.02 | 0.40 |
| 3 | HC | 61 | F |  |  |  |  |  | 139 | 203 | 79 | 47 | 3.4 | 59 | 59.4 | 10.40 | 1.42 | 8.86 | 6.38 | 32.65 | 20.39 | 7.76 | 7.27 | 1.55 | 0.23 | 1.97 | 0.33 |
| 4 | HC | 60 | F |  |  |  |  |  | 143 | 88 | 80 | 49 | 7.3 | 79 | 60.9 | 7.99 | 1.56 | 5.80 | 4.92 | 36.21 | 17.64 | 6.80 | 12.02 | 1.61 | 0.29 | 3.02 | 0.38 |
| 5 | HC | 61 | M |  |  |  |  |  | 145 | 187 | 70 | 45 | 6 | 86 | 76.2 | 8.81 | 2.37 | 5.84 | 5.94 | 39.15 | 18.22 | 4.67 | 8.49 | 3.06 | 0.32 | 1.64 | 0.66 |
| 6 | HC | 73 | M |  |  |  |  |  | 128 | 291 | 81 | 47 | 6.2 | 74 | 59.8 | 8.32 | 1.24 | 6.51 | 5.51 | 39.49 | 21.30 | 5.57 | 7.23 | 1.91 | 0.70 | 1.09 | 0.00 |
| 7 | HC | 68 | F |  |  |  |  |  | 138 | 224 | 77 | 45 | 5.9 | 72 | 62.8 | 9.37 | 1.07 | 6.09 | 3.58 | 41.77 | 17.43 | 5.99 | 9.76 | 1.72 | 0.32 | 1.76 | 0.43 |
| 8 | HC | 68 | F |  |  |  |  |  | 144 | 244 | 82 | 48 | 5.9 | 64 | 72.4 | 10.45 | 2.00 | 8.25 | 5.74 | 32.42 | 21.76 | 7.93 | 7.87 | 0.88 | 0.23 | 1.41 | 0.00 |
| 9 | HC | 62 | M |  |  |  |  |  | 155 | 188 | 74 | 43 | 6.4 | 75 | 51.2 | 9.84 | 1.84 | 6.12 | 7.77 | 37.64 | 18.30 | 6.73 | 5.45 | 4.33 | 0.32 | 0.67 | 0.48 |
| 10 | HC | 69 | M |  |  |  |  |  | 145 | 156 | 75 | 46 | 5.2 | 65 | 60.9 | 9.58 | 1.60 | 7.86 | 3.97 | 37.84 | 20.32 | 8.83 | 6.00 | 2.23 | 0.90 | 0.51 | 0.00 |
| 11 | HC | 56 | M |  |  |  |  |  | 164 | 147 | 69 | 45 | 7.7 | 83 | 72.6 | 6.27 | 1.26 | 5.23 | 4.58 | 41.94 | 20.70 | 5.51 | 5.58 | 5.63 | 0.28 | 1.40 | 0.98 |
| 12 | HC | 55 | F |  |  |  |  |  | 133 | 191 | 72 | 45 | 6.3 | 60 | 63.2 | 8.67 | 1.93 | 8.41 | 5.52 | 36.83 | 19.21 | 5.49 | 8.30 | 1.54 | 0.36 | 2.76 | 0.00 |
| 13 | HC | 74 | F |  |  |  |  |  | 133 | 167 | 78 | 47 | 6.2 | 73 | 61.5 | 12.98 | 2.42 | 5.61 | 5.02 | 36.86 | 17.74 | 6.30 | 9.28 | 0.38 | 0.12 | 2.06 | 0.00 |
| 14 | HC | 63 | M |  |  |  |  |  | 135 | 140 | 73 | 47 | 6.2 | 62 | 75.7 | 11.28 | 1.79 | 7.86 | 4.82 | 38.26 | 17.50 | 5.12 | 7.12 | 2.76 | 0.26 | 1.99 | 0.55 |
| 15 | HC | 55 | F |  |  |  |  |  | 136 | 172 | 76 | 48 | 5.6 | 60 | 71.4 | 8.63 | 1.52 | 7.07 | 5.26 | 36.94 | 19.62 | 6.46 | 9.59 | 1.25 | 0.14 | 2.46 | 0.27 |
| 16 | HC | 54 | M |  |  |  |  |  | 142 | 256 | 76 | 46 | 5.5 | 85 | 59.7 | 7.39 | 1.28 | 6.91 | 5.92 | 37.77 | 21.46 | 5.23 | 7.20 | 3.50 | 0.29 | 1.57 | 0.64 |
| 17 | HC | 62 | M |  |  |  |  |  | 157 | 206 | 76 | 49 | 5.5 | 83 | 59.3 | 5.74 | 1.17 | 5.49 | 5.91 | 40.74 | 22.59 | 5.83 | 6.24 | 2.85 | 0.42 | 1.47 | 0.75 |
| 18 | HC | 61 | F |  |  |  |  |  | 134 | 246 | 84 | 48 | 3.8 | 51 | 49.2 | 8.87 | 1.14 | 6.30 | 5.12 | 39.14 | 17.21 | 6.57 | 8.16 | 4.07 | 0.38 | 1.52 | 0.67 |
| 19 | HC | 55 | F |  |  |  |  |  | 147 | 280 | 79 | 50 | 6.4 | 62 | 65.3 | 7.71 | 1.21 | 6.10 | 4.38 | 40.84 | 18.79 | 8.30 | 6.17 | 3.66 | 0.37 | 1.17 | 0.62 |
| 20 | HC | 64 | F |  |  |  |  |  | 149 | 182 | 83 | 47 | 6.4 | 63 | 52.8 | 7.80 | 1.07 | 5.22 | 5.08 | 37.88 | 20.89 | 5.96 | 9.33 | 2.05 | 0.41 | 2.21 | 0.45 |
| 21 | HC | 55 | M |  |  |  |  |  | 156 | 225 | 71 | 44 | 6.4 | 88 | 72.3 | 6.38 | 1.18 | 5.29 | 4.66 | 43.04 | 17.87 | 7.87 | 8.50 | 2.31 | 0.26 | 1.49 | 0.44 |
| 22 | HC | 63 | F |  |  |  |  |  | 132 | 188 | 74 | 44 | 6.3 | 69 | 59.7 | 6.06 | 0.97 | 6.91 | 7.53 | 38.62 | 20.69 | 5.74 | 7.69 | 2.54 | 0.26 | 1.73 | 0.57 |
| 23 | HC | 59 | F |  |  |  |  |  | 141 | 201 | 71 | 43 | 5.4 | 76 | 75.5 | 6.17 | 1.06 | 7.20 | 7.35 | 38.18 | 21.99 | 4.54 | 7.65 | 1.88 | 0.31 | 2.24 | 0.68 |
| 24 | HC | 54 | M |  |  |  |  |  | 151 | 201 | 73 | 49 | 6.6 | 68 | 64.7 | 8.84 | 1.44 | 5.92 | 4.26 | 41.75 | 19.18 | 4.32 | 8.51 | 2.18 | 0.26 | 1.91 | 0.40 |
| 25 | HC | 52 | M |  |  |  |  |  | 152 | 247 | 73.8 | 45.4 | 5.5 | 78 | 59.4 | 6.02 | 0.89 | 5.62 | 5.05 | 37.90 | 23.92 | 6.71 | 7.27 | 3.52 | 0.40 | 1.17 | 0.53 |
| 26 | HC | 54 | F |  |  |  |  |  | 150 | 102 | 74.4 | 47.4 | 6.2 | 90 | 61.8 | 6.71 | 1.32 | 5.52 | 5.20 | 39.31 | 19.62 | 8.90 | 7.65 | 2.51 | 0.25 | 1.63 | 0.56 |
| 27 | HC | 56 | M |  |  |  |  |  | 160 | 118 | 80.1 | 50.1 | 4.7 | 57 | 52.3 | 8.81 | 2.19 | 5.36 | 4.64 | 34.77 | 23.77 | 9.07 | 5.96 | 1.79 | 0.46 | 1.19 | 0.46 |
| 28 | HC | 57 | F |  |  |  |  |  | 150 | 260 | 78.5 | 48.1 | 4.8 | 58 | 50.9 | 9.36 | 0.74 | 7.14 | 7.31 | 38.10 | 22.00 | 5.01 | 3.20 | 4.93 | 0.33 | 0.74 | 0.57 |
| 29 | HC | 55 | M |  |  |  |  |  | 151 | 190 | 79.8 | 50 | 7.5 | 65 | 62.8 | 8.39 | 1.17 | 7.39 | 6.00 | 35.09 | 21.26 | 6.78 | 8.39 | 2.61 | 0.26 | 1.52 | 0.48 |
| 30 | HC | 57 | M |  |  |  |  |  | 142 | 283 | 82.1 | 52.8 | 5.5 | 82 | 85.8 | 5.76 | 0.87 | 4.20 | 4.67 | 41.80 | 18.39 | 5.06 | 10.30 | 5.41 | 0.39 | 1.73 | 0.74 |

| No. | group | age | gender | LC | ISS stage | chemo | SPE | IFE | Hb | PLT | TP | ALB | BUN | CREA | SA | P1 | P2 | P3 | P4 | P5 | P6 | P7 | P8 | P9 | P10 | P11 | P12 |
| --- | --- | --- | --- | --- | --- | --- | --- | --- | --- | --- | --- | --- | --- | --- | --- | --- | --- | --- | --- | --- | --- | --- | --- | --- | --- | --- | --- |
| 31 | HC | 53 | M |  |  |  |  |  | 157 | 207 | 79.5 | 46.5 | 5 | 88 | 64.8 | 7.28 | 0.85 | 5.34 | 6.66 | 36.22 | 22.52 | 7.43 | 6.97 | 3.17 | 0.46 | 1.47 | 0.62 |
| 32 | HC | 58 | M |  |  |  |  |  | 155 | 175 | 76.5 | 46.2 | 6.2 | 86 | 56.9 | 7.90 | 1.66 | 6.87 | 6.08 | 34.91 | 23.22 | 8.29 | 5.85 | 2.61 | 0.39 | 1.03 | 0.47 |
| 33 | HC | 71 | M |  |  |  |  |  |  |  | 73.2 | 43.9 | 4.6 | 78 | 67 | 8.26 | 1.26 | 5.75 | 6.81 | 39.13 | 20.09 | 5.68 | 7.27 | 1.92 | 0.46 | 1.72 | 0.73 |
| 34 | HC | 59 | M |  |  |  |  |  | 147 | 266 | 76.2 | 48.8 | 6.2 | 74 | 72 | 7.35 | 0.66 | 7.18 | 6.54 | 40.64 | 20.50 | 5.05 | 4.45 | 4.92 | 0.30 | 1.06 | 0.82 |
| 35 | HC | 55 | M |  |  |  |  |  | 148 | 166 | 71.1 | 49.6 | 6.7 | 79 | 59.1 | 5.85 | 1.17 | 4.53 | 3.99 | 46.61 | 16.20 | 4.19 | 10.55 | 2.58 | 0.36 | 2.52 | 0.44 |
| 36 | HC | 58 | F |  |  |  |  |  | 143 | 180 | 74.8 | 47 | 5.5 | 53 | 51 | 5.44 | 1.04 | 5.85 | 5.01 | 40.64 | 19.55 | 5.59 | 11.34 | 0.91 | 0.27 | 2.98 | 0.16 |
| 37 | HC | 60 | M |  |  |  |  |  | 154 | 191 | 77.5 | 49.8 | 6.6 | 80 | 70.2 | 7.04 | 1.64 | 6.72 | 5.98 | 39.45 | 21.04 | 7.28 | 5.30 | 3.11 | 0.34 | 0.89 | 0.53 |
| 38 | HC | 55 | F |  |  |  |  |  | 144 | 316 | 83 | 48.1 | 3.2 | 60 | 67.9 | 5.70 | 0.46 | 7.75 | 6.06 | 32.74 | 28.84 | 6.92 | 7.19 | 1.22 | 0.26 | 1.60 | 0.20 |
| 39 | HC | 59 | M |  |  |  |  |  | 149 | 177 | 77 | 50.6 | 7.7 | 88 | 53.2 | 6.89 | 1.13 | 5.34 | 4.31 | 44.92 | 13.93 | 2.99 | 10.92 | 4.83 | 0.38 | 2.49 | 0.85 |
| 40 | HC | 48 | F |  |  |  |  |  | 130 | 267 | 74.2 | 46.6 | 5.4 | 64 | 60 | 5.70 | 1.11 | 5.09 | 9.18 | 40.92 | 19.94 | 6.23 | 6.20 | 2.95 | 0.26 | 1.32 | 0.56 |
| 41 | HC | 63 | M |  |  |  |  |  | 164 | 172 | 73.2 | 46.3 | 5.4 | 86 | 65.4 | 11.46 | 1.49 | 7.86 | 8.00 | 36.58 | 17.68 | 4.52 | 6.91 | 2.57 | 0.37 | 1.32 | 0.39 |
| 42 | HC | 54 | M |  |  |  |  |  | 147 | 235 | 70.2 | 46.9 | 7.8 | 74 | 62.6 | 3.29 | 0.60 | 5.30 | 5.10 | 40.53 | 22.58 | 5.15 | 11.98 | 0.96 | 0.23 | 2.65 | 0.25 |
| 43 | LCMM | 53 | F | L | 3 | C | N | N | 71 | 261 | 66.6 | 39.1 |  |  | 131.1 | 6.33 | 0.47 | 3.53 | 4.56 | 46.26 | 10.83 | 0.98 | 15.74 | 4.84 | 0.54 | 4.73 | 1.19 |
| 44 | LCMM | 56 | M | K | 3 | C | P | P | 82 | 139 | 51.6 | 32.5 | 9.6 | 269 | 107.1 | 2.24 | 0.38 | 1.23 | 3.21 | 52.22 | 10.50 | 1.29 | 8.29 | 14.46 | 0.44 | 2.46 | 3.27 |
| 45 | LCMM | 60 | F | K | 1 | C | N | N | 102 | 73 | 67.5 | 44 | 4.2 | 54 | 59.5 | 9.55 | 1.64 | 5.80 | 5.67 | 42.11 | 18.07 | 3.22 | 8.91 | 2.35 | 0.21 | 2.00 | 0.46 |
| 46 | LCMM | 65 | F | L | 1 | C | N | N | 126 | 179 | 64.7 | 40.7 | 7.7 | 93 | 74.5 | 11.22 | 0.72 | 6.68 | 5.87 | 38.57 | 20.19 | 3.02 | 8.28 | 2.51 | 0.27 | 2.09 | 0.59 |
| 47 | LCMM | 51 | F | K | 1 | C | N | N | 120 | 69 | 54.6 | 40.2 | 4.3 | 86 | 62.5 | 3.75 | 0.77 | 2.84 | 3.50 | 52.16 | 16.06 | 3.10 | 11.72 | 2.02 | 0.29 | 3.25 | 0.53 |
| 48 | LCMM | 57 | M | L | 3 | U | N | P | 46 | 124 | 68.3 | 39.7 | 8.2 | 138 | 91.7 | 3.33 | 0.33 | 1.60 | 1.89 | 21.82 | 44.87 | 16.09 | 4.99 | 2.48 | 0.25 | 1.62 | 0.75 |
| 49 | LCMM | 60 | M | K | 2 | C | N | N | 91 | 162 | 58.3 | 34.5 | 4.8 | 69 | 98.3 | 8.95 | 0.71 | 4.21 | 4.66 | 42.25 | 15.99 | 3.91 | 6.44 | 8.66 | 0.60 | 1.83 | 1.80 |
| 50 | LCMM | 65 | M | K | 1 | C | P | P | 134 | 213 | 62.6 | 37.9 | 4.2 | 53 | 71.1 | 8.29 | 1.65 | 4.44 | 4.59 | 40.19 | 17.70 | 5.24 | 12.11 | 1.87 | 0.33 | 3.04 | 0.54 |
| 51 | LCMM | 63 | M | L | 3 | C | P | P |  |  | 44.7 | 26.7 | 13.1 | 234 | 78.2 | 4.04 | 0.64 | 2.60 | 3.97 | 49.16 | 12.52 | 1.41 | 10.38 | 9.55 | 0.96 | 2.73 | 2.04 |
| 52 | LCMM | 64 | F | L | 3 | U | N | N | 111 | 159 | 69.6 | 49.7 | 18.3 | 210 | 64 | 12.62 | 1.69 | 5.39 | 5.47 | 44.06 | 14.46 | 1.90 | 7.47 | 3.10 | 0.19 | 2.75 | 0.89 |
| 53 | LCMM | 57 | M | K | 1 | C | N | N | 139 | 132 | 58.6 | 40 | 3.6 | 57 | 62.2 | 9.64 | 0.95 | 5.86 | 5.38 | 42.54 | 18.31 | 2.60 | 7.66 | 3.92 | 0.45 | 1.95 | 0.73 |
| 54 | LCMM | 48 | F | K | 1 | C | N | N | 161 | 163 | 59.7 | 46 | 2.4 | 45 | 51.4 | 9.24 | 1.13 | 4.35 | 4.46 | 46.95 | 17.04 | 1.51 | 10.27 | 0.92 | 0.24 | 3.59 | 0.30 |
| 55 | LCMM | 56 | F | L | 1 | C | N | N | 145 | 233 | 68.3 | 46.1 | 2 | 40 | 72 | 5.45 | 0.68 | 4.90 | 4.77 | 42.02 | 21.58 | 3.54 | 9.03 | 4.48 | 0.36 | 2.29 | 0.90 |
| 56 | LCMM | 57 | M | K | 3 | C | N | N | 77 | 190 | 57.7 | 39.1 | 19 | 246 | 63.4 | 6.73 | 0.91 | 4.38 | 5.05 | 40.01 | 17.21 | 1.43 | 12.49 | 6.06 | 0.72 | 3.69 | 1.32 |
| 57 | LCMM | 64 | M | L | 2 | U | N | N | 151 | 202 | 68.8 | 44.8 | 5.3 | 120 | 58.7 | 15.68 | 3.31 | 5.20 | 5.67 | 37.98 | 13.77 | 3.71 | 9.25 | 2.30 | 0.23 | 2.44 | 0.46 |
| 58 | LCMM | 77 | M | L | 3 | U | N | N | 58 | 78 | 58.6 | 37.8 | 28.5 | 732 | 73.1 | 7.95 | 0.99 | 3.18 | 4.29 | 47.32 | 12.81 | 4.02 | 7.12 | 7.83 | 0.60 | 2.03 | 1.87 |
| 59 | LCMM | 44 | F | K | 3 | C | P | P | 57 | 114 | 57.7 | 40.1 | 4.9 | 59 | 83 | 5.82 | 0.84 | 3.42 | 4.11 | 46.65 | 13.33 | 2.87 | 15.82 | 2.17 | 0.29 | 4.12 | 0.55 |
| 60 | LCMM | 59 | M | K | 3 | C | N | N | 81 | 356 | 46.5 | 29.4 | 17 | 166 | 92.2 | 5.10 | 0.50 | 1.79 | 3.84 | 49.65 | 11.42 | 1.52 | 8.40 | 10.24 | 0.92 | 3.45 | 3.16 |

| No. | group | age | gender | LC | ISS stage | chemo | SPE | IFE | Hb | PLT | TP | ALB | BUN | CREA | SA | P1 | P2 | P3 | P4 | P5 | P6 | P7 | P8 | P9 | P10 | P11 | P12 |
| --- | --- | --- | --- | --- | --- | --- | --- | --- | --- | --- | --- | --- | --- | --- | --- | --- | --- | --- | --- | --- | --- | --- | --- | --- | --- | --- | --- |
| 61 | LCMM | 68 | F | K | 3 | C | N | P | 78 | 54 | 48.8 | 31.1 | 8.1 | 101 | 89 | 9.03 | 0.56 | 2.19 | 3.53 | 46.59 | 8.47 | 1.09 | 19.00 | 0.45 | 0.21 | 8.45 | 0.42 |
| 62 | LCMM | 76 | M | K | 3 | C | N | P | 109 | 258 | 57.2 | 34.9 | 24.6 | 444 | 73.8 | 11.83 | 0.71 | 4.64 | 4.56 | 46.80 | 13.49 | 1.92 | 5.35 | 7.38 | 0.58 | 1.29 | 1.45 |
| 63 | LCMM | 76 | F | K | 3 | C | N | P | 78 | 34 | 60.3 | 42.6 | 14.8 | 183 | 72.1 | 7.65 | 0.86 | 2.59 | 3.48 | 53.24 | 11.92 | 1.32 | 9.93 | 3.35 | 0.27 | 4.18 | 1.20 |
| 64 | LCMM | 23 | M | L | 3 | U | P | P | 76 | 210 | 62.8 | 42 | 12.6 | 279 | 76.3 | 5.36 | 0.89 | 1.24 | 3.00 | 52.76 | 12.37 | 3.44 | 9.75 | 6.34 | 0.56 | 2.73 | 1.56 |
| 65 | LCMM | 76 | M | K | 3 | C | P | P | 87 | 82 | 50.7 | 35 | 21.5 | 1192 | 63.3 | 6.39 | 1.23 | 1.81 | 3.22 | 53.95 | 12.64 | 1.31 | 5.77 | 10.03 | 0.68 | 1.21 | 1.76 |
| 66 | LCMM | 63 | M | L | 3 | U | N | P | 109 | 304 | 61.2 | 40.9 | 9.3 | 153 | 74.8 | 3.81 | 0.47 | 1.93 | 3.65 | 56.99 | 11.20 | 0.95 | 12.03 | 3.26 | 0.31 | 4.39 | 1.01 |
| 67 | LCMM | 34 | M | L | 3 | C | N | P | 111 | 226 | 58.9 | 40.6 | 11.8 | 168 | 69.7 | 3.85 | 0.71 | 2.87 | 3.80 | 51.10 | 13.99 | 1.61 | 10.31 | 6.17 | 0.60 | 3.32 | 1.66 |
| 68 | LCMM | 34 | F | K | 1 | C | N | P | 109 | 243 | 62.2 | 39.4 | 6.3 | 87 | 70.9 | 4.62 | 0.58 | 3.16 | 4.32 | 53.09 | 15.09 | 1.31 | 10.13 | 3.58 | 0.28 | 2.91 | 0.92 |
| 69 | LCMM | 69 | F | L | 3 | C | N | N | 104 | 101 | 63.1 | 44.7 | 8.9 | 150 | 70.4 | 5.05 | 0.45 | 2.48 | 3.19 | 50.23 | 10.97 | 1.08 | 18.03 | 1.54 | 0.29 | 6.24 | 0.44 |
| 70 | LCMM | 63 | F | K | 3 | C | P | P | 74 | 20 | 76.5 | 51.6 | 8.8 | 126 | 80.7 | 3.23 | 0.36 | 2.01 | 3.69 | 59.52 | 14.58 | 1.34 | 8.28 | 2.86 | 0.34 | 2.86 | 0.93 |
| 71 | LCMM | 35 | M | L | 3 | C | N | N | 69 | 289 | 58.2 | 37.1 | 4.9 | 196 | 71 | 3.57 | 0.52 | 3.00 | 4.34 | 51.28 | 13.00 | 1.41 | 8.84 | 8.23 | 0.45 | 3.04 | 2.32 |
| 72 | LCMM | 72 | F | L | 3 | C | P | P | 100 | 40 | 55.5 | 33.3 | 8.9 | 355 | 82.2 | 6.67 | 0.72 | 1.84 | 4.11 | 51.25 | 14.73 | 2.03 | 6.50 | 7.55 | 0.97 | 1.88 | 1.74 |
| 73 | LCMM | 69 | M | L | 3 | C | N | P | 97 | 268 | 68.6 | 37.8 | 20.9 | 397 | 117.6 | 8.56 | 0.42 | 2.97 | 3.35 | 54.21 | 10.04 | 1.46 | 6.52 | 8.14 | 0.37 | 1.92 | 2.04 |
| 74 | LCMM | 55 | M | L | 3 | U | N | P | 77 | 149 | 68.5 | 38.4 | 8.5 | 210 | 84.7 | 7.26 | 0.87 | 3.87 | 4.24 | 46.65 | 13.54 | 2.13 | 9.93 | 7.15 | 0.68 | 2.32 | 1.36 |
| 75 | LCMM | 55 | M | K | 3 | U | N | P | 114 | 204 | 63.3 | 42.8 | 7.8 | 173 | 61.7 | 8.59 | 1.79 | 3.86 | 4.55 | 47.88 | 13.30 | 2.16 | 8.59 | 4.55 | 0.53 | 2.82 | 1.38 |
| 76 | LCMM | 64 | M | K | 1 | U | N | N | 139 | 266 | 54.6 | 35.1 | 10.7 | 46 | 64.1 | 6.72 | 1.01 | 2.64 | 4.58 | 48.87 | 10.64 | 1.36 | 17.33 | 1.48 | 0.35 | 4.39 | 0.62 |
| 77 | LCMM | 71 | F | L | 3 | U | P | P | 63 | 159 | 77.3 | 36.7 | 12.3 | 169 | 87.1 | 7.93 | 1.24 | 2.88 | 2.88 | 44.28 | 13.33 | 9.53 | 7.86 | 4.91 | 0.59 | 2.88 | 1.67 |
| 78 | LCMM | 65 | M | L | 3 | U | P | P | 89 | 186 | 54.3 | 37.2 | 5.9 | 70 | 61.7 | 6.17 | 1.31 | 2.99 | 3.84 | 47.10 | 11.60 | 1.96 | 8.61 | 11.13 | 0.65 | 2.39 | 2.25 |
| 79 | LCMM | 51 | M | K | 1 | C | P | P | 131 | 112 | 69 | 44.5 | 6.2 | 46 | 57.8 | 12.00 | 1.07 | 6.23 | 5.21 | 42.30 | 17.71 | 2.63 | 7.65 | 2.61 | 0.23 | 1.80 | 0.55 |
| 80 | LCMM | 58 | M | L | 1 | U | N | N | 97 | 248 | 82.7 | 42.4 | 4.7 | 52 | 78.3 | 5.12 | 0.90 | 3.28 | 3.96 | 50.48 | 12.27 | 1.90 | 12.72 | 4.61 | 0.31 | 3.24 | 1.20 |
| 81 | LCMM | 46 | M | L | 2 | C | N | P | 146 | 226 | 66.7 | 41.4 | 4.5 | 90 | 76.1 | 7.08 | 0.69 | 4.26 | 3.91 | 48.36 | 17.96 | 3.05 | 7.20 | 3.91 | 0.92 | 1.96 | 0.69 |
| 82 | LCMM | 68 | M | L | 3 | U | N | N | 90 | 220 | 57.2 | 36.6 | 23.3 | 532 | 62.4 | 4.50 | 0.76 | 4.32 | 4.25 | 50.62 | 14.38 | 3.30 | 12.46 | 1.87 | 0.24 | 2.95 | 0.35 |
| 83 | LCMM | 60 | F | L | 3 | U | P | P | 111 | 150 | 62.9 | 44.3 | 12.6 | 167 | 53.9 | 8.32 | 1.32 | 3.23 | 3.47 | 52.04 | 12.03 | 1.66 | 8.03 | 5.66 | 0.26 | 2.58 | 1.41 |
| 84 | LCMM | 49 | M | L | 3 | C | N | P | 64 | 6 | 48.4 | 26.3 | 7.5 | 69 | 85.9 | 2.83 | 0.40 | 2.07 | 2.94 | 50.01 | 12.63 | 1.38 | 15.97 | 5.08 | 0.69 | 4.61 | 1.40 |
| 85 | IgGMM | 69 | M | L | 2 | C | P | P | 114 | 163 | 72.1 | 41.7 | 2.6 | 68 |  | 57.70 | 4.22 | 15.30 | 6.23 | 8.64 | 4.98 | 0.56 | 1.48 | 0.47 | 0.07 | 0.25 | 0.09 |
| 86 | IgGMM | 75 | F | L | 1 | U | P | P | 115 | 199 | 69.5 | 44.2 | 5.9 | 72 |  | 15.60 | 3.59 | 11.97 | 6.46 | 30.66 | 15.65 | 1.98 | 8.30 | 2.31 | 0.59 | 2.37 | 0.52 |
| 87 | IgGMM | 71 | F | K | 2 | U | P | P | 124 | 129 | 94.3 | 27.2 | 7.7 | 88 |  | 3.92 | 0.30 | 11.04 | 5.25 | 41.92 | 23.40 | 1.43 | 7.72 | 2.36 | 0.34 | 1.84 | 0.49 |
| 88 | IgGMM | 42 | M | L | 3 | C | P | P | 133 | 192 | 57.4 | 42.3 | 4.1 | 62 |  | 3.77 | 0.59 | 2.18 | 3.59 | 43.43 | 16.78 | 7.60 | 9.64 | 7.31 | 0.33 | 3.03 | 1.74 |
| 89 | IgGMM | 91 | F | K | 2 | C | P | P | 104 | 276 | 54 | 28.5 | 5.6 | 48 |  | 16.84 | 0.12 | 34.16 | 11.58 | 9.35 | 23.16 | 0.30 | 1.68 | 1.82 | 0.14 | 0.46 | 0.42 |
| 90 | IgGMM | 61 | M | L | 3 | C | P | P | 143 | 188 | 67.6 | 43.2 | 5.6 | 64 |  | 16.39 | 0.15 | 33.83 | 11.47 | 9.53 | 23.54 | 0.29 | 1.75 | 1.94 | 0.16 | 0.49 | 0.47 |

Continued S3 Table

| No. | group | age | gender | LC | ISS stage | chemo | SPE | IFE | Hb | PLT | TP | ALB | BUN | CREA | SA | P1 | P2 | P3 | P4 | P5 | P6 | P7 | P8 | P9 | P10 | P11 | P12 |
| --- | --- | --- | --- | --- | --- | --- | --- | --- | --- | --- | --- | --- | --- | --- | --- | --- | --- | --- | --- | --- | --- | --- | --- | --- | --- | --- | --- |
| 91 | IgGMM | 61 | M | K | 1 | U | P | P | 104 | 87 | 61 | 40 | 10.9 | 82 |  | 9.21 | 0.78 | 7.03 | 5.36 | 45.24 | 17.19 | 2.85 | 5.37 | 4.36 | 0.45 | 1.28 | 0.90 |
| 92 | IgGMM | 75 | F | K | 2 | C | P | P | 96 | 153 | 55.6 | 34.2 | 3.5 | 45 |  | 3.93 | 0.57 | 2.98 | 3.18 | 48.25 | 19.21 | 4.15 | 12.62 | 1.06 | 0.25 | 3.52 | 0.29 |
| 93 | IgGMM | 60 | M | L | 1 | C | P | P | 96 | 108 | 57.7 | 36 |  |  |  | 11.59 | 1.93 | 10.06 | 6.49 | 32.64 | 22.89 | 5.20 | 5.91 | 1.18 | 0.30 | 1.55 | 0.26 |
| 94 | IgGMM | 55 | F | L | 2 | C | P | P | 130 | 108 | 73.6 | 46.6 | 3.4 | 53 |  | 5.96 | 0.99 | 4.37 | 4.85 | 47.67 | 14.41 | 2.73 | 10.15 | 5.43 | 0.34 | 2.17 | 0.92 |
| 95 | IgGMM | 75 | M | K | 1 | C | P | P | 101 | 204 | 72.3 | 35.6 | 4 | 54 |  | 7.28 | 0.56 | 4.75 | 4.42 | 48.57 | 16.13 | 1.61 | 8.81 | 4.93 | 0.50 | 1.64 | 0.79 |
| 96 | IgGMM | 72 | F | K | 3 | C | P | P | 64 | 104 | 106.9 | 22.2 | 4.9 | 66 |  | 21.55 | 2.53 | 24.87 | 9.83 | 19.38 | 12.80 | 1.25 | 3.76 | 1.94 | 0.13 | 1.33 | 0.63 |
| 97 | IgGMM | 53 | M | L | 2 | C | P | P | 122 | 224 | 62.2 | 37.5 | 4.7 | 60 |  | 51.78 | 15.98 | 8.46 | 5.79 | 8.81 | 4.90 | 0.53 | 1.03 | 2.10 | 0.09 | 0.20 | 0.35 |
| 98 | IgGMM | 75 | M | K | 1 | C | N | N | 123 | 193 | 58.2 | 40.2 | 7.1 | 66 |  | 8.84 | 0.63 | 9.50 | 6.03 | 41.55 | 16.28 | 2.11 | 7.73 | 3.47 | 0.25 | 2.59 | 1.02 |
| 99 | IgGMM | 77 | M | L | 3 | C | P | P | 71 | 164 | 122.8 | 24.3 | 4.5 | 85 |  | 7.76 | 2.71 | 9.57 | 4.93 | 39.14 | 17.56 | 3.23 | 8.10 | 3.22 | 0.50 | 2.40 | 0.87 |
| 100 | IgGMM | 53 | M | L | 1 | C | P | P | 118 | 163 | 58 | 39.1 | 3.2 | 87 |  | 6.51 | 0.30 | 15.81 | 5.60 | 33.42 | 24.13 | 1.08 | 7.95 | 2.73 | 0.12 | 1.79 | 0.55 |
| 101 | IgGMM | 54 | M | L | 1 | U | P | P | 142 | 177 | 65.4 | 39.3 | 3.2 | 64 |  | 12.76 | 0.55 | 17.50 | 8.34 | 7.34 | 46.53 | 3.10 | 1.61 | 1.27 | 0.14 | 0.53 | 0.34 |
| 102 | IgGMM | 55 | F | K | 2 | C | P | P | 121 | 125 | 62.3 | 45.1 | 7.4 | 75 |  | 23.40 | 0.18 | 18.04 | 6.18 | 4.43 | 43.57 | 2.53 | 0.95 | 0.32 | 0.05 | 0.24 | 0.09 |
| 103 | IgGMM | 53 | M | K | 2 | C | P | P | 120 | 177 | 76.4 | 49.3 | 4.1 | 43 |  | 6.56 | 1.14 | 9.01 | 5.13 | 41.19 | 18.86 | 2.88 | 9.98 | 1.45 | 0.24 | 3.09 | 0.47 |
| 104 | IgGMM | 54 | F | K | 3 | C | P | P | 133 | 112 | 67.8 | 47.1 | 4.6 | 57 |  | 17.83 | 2.08 | 13.39 | 7.18 | 32.21 | 15.27 | 2.07 | 6.62 | 1.21 | 0.16 | 1.72 | 0.26 |
| 105 | IgGMM | 67 | M | K | 1 | U | P | P | 107 | 115 | 58.9 | 37.8 | 3.8 | 62 |  | 8.44 | 0.72 | 4.59 | 5.47 | 43.96 | 18.18 | 2.18 | 11.19 | 1.70 | 0.23 | 2.96 | 0.38 |
| 106 | IgGMM | 81 | F | K | 3 | C | P | P | 74 | 92 | 94 | 27.5 | 6.9 | 71 |  | 14.10 | 2.95 | 8.45 | 4.54 | 28.86 | 14.51 | 15.87 | 6.16 | 1.70 | 0.19 | 2.17 | 0.51 |
| 107 | IgGMM | 84 | F | K | 2 | U | P | P | 136 | 311 | 60 | 32.9 | 4.9 | 65 |  | 50.17 | 2.87 | 3.44 | 4.92 | 19.80 | 8.21 | 0.97 | 2.89 | 4.04 | 0.78 | 0.86 | 1.03 |
| 108 | IgGMM | 58 | M | K | 1 | C | P | P | 107 | 121 | 65.9 | 37.9 | 8.3 | 70 |  | 11.32 | 1.60 | 7.38 | 6.23 | 35.51 | 20.35 | 7.00 | 6.81 | 0.93 | 0.36 | 2.17 | 0.33 |
| 109 | IgGMM | 63 | M | K | 2 | C | P | P | 143 | 122 | 77.8 | 44.3 | 5.2 | 105 |  | 6.99 | 0.93 | 5.20 | 4.06 | 46.66 | 17.35 | 2.76 | 9.53 | 3.58 | 0.28 | 1.94 | 0.72 |
| 110 | IgGMM | 67 | F | K | 3 | U | P | P | 66 | 103 | 98.5 | 24.1 | 7.5 | 106 |  | 9.92 | 1.37 | 7.69 | 5.67 | 28.77 | 26.13 | 10.05 | 5.51 | 2.23 | 0.39 | 1.69 | 0.58 |
| 111 | IgGMM | 42 | F | K | 3 | C | P | P | 51 | 14 | 113.9 | 18.4 | 3.9 | 46 |  | 10.70 | 1.29 | 8.62 | 5.91 | 37.59 | 20.81 | 5.31 | 7.48 | 0.54 | 0.16 | 1.49 | 0.11 |
| 112 | IgGMM | 60 | F | L | 2 | C | P | P | 57 | 265 | 73.2 | 31.8 | 8.8 | 86 |  | 7.61 | 0.69 | 7.64 | 5.73 | 39.98 | 17.82 | 2.13 | 7.03 | 7.61 | 0.53 | 1.80 | 1.44 |
| 113 | IgGMM | 91 | M | K | 1 | C | P | P | 104 | 132 | 59.5 | 37.7 | 10 | 109 |  | 14.72 | 2.09 | 7.12 | 6.96 | 36.36 | 17.47 | 5.16 | 7.21 | 0.92 | 0.17 | 1.60 | 0.23 |
| 114 | IgGMM | 75 | F | K | 1 | C | N | P | 137 | 162 | 71 | 41.5 | 5.3 | 54 |  | 12.06 | 0.99 | 12.74 | 5.87 | 34.12 | 21.35 | 2.66 | 5.56 | 2.32 | 0.17 | 1.62 | 0.54 |
| 115 | IgGMM | 75 | M | K | 2 | U | P | P | 115 | 206 | 52.1 | 37.1 | 6.4 | 78 |  | 4.99 | 1.10 | 23.83 | 8.61 | 19.49 | 31.54 | 2.80 | 3.08 | 2.82 | 0.19 | 0.87 | 0.70 |
| 116 | IgGMM | 52 | M | L | 3 | C | P | P | 149 | 130 | 65.2 | 44.3 | 5.5 | 61 |  | 19.72 | 1.73 | 12.86 | 6.50 | 27.25 | 9.60 | 10.43 | 6.09 | 3.19 | 0.21 | 1.68 | 0.75 |
| 117 | IgGMM | 58 | M | L | 2 | C | P | P | 136 | 124 | 64.7 | 39.8 | 4 | 55 |  | 12.60 | 1.14 | 13.08 | 6.43 | 35.50 | 16.59 | 1.68 | 5.37 | 4.77 | 0.60 | 1.35 | 0.88 |
| 118 | IgGMM | 56 | F | L | 3 | C | P | P | 70 | 118 | 101 | 25.8 | 6.6 | 47 |  | 13.58 | 0.31 | 3.85 | 4.41 | 39.06 | 21.76 | 1.16 | 4.22 | 6.24 | 1.25 | 1.82 | 2.33 |
| 119 | IgGMM | 55 | M | L | 3 | C | P | P | 122 | 165 | 53.3 | 35.6 | 6 | 94 |  | 22.97 | 2.02 | 16.82 | 7.76 | 17.53 | 11.12 | 12.87 | 4.75 | 2.27 | 0.19 | 1.17 | 0.54 |
| 120 | IgGMM | 58 | M | L | 3 | C | P | P | 125 | 422 | 90.1 | 31.7 | 6.4 | 74 |  | 3.40 | 0.42 | 0.84 | 3.78 | 62.20 | 12.26 | 0.92 | 7.17 | 6.14 | 0.41 | 1.54 | 0.92 |

Continued S3 Table

| No. | group | age | gender | LC | ISS stage | chemo | SPE | IFE | Hb | PLT | TP | ALB | BUN | CREA | SA | P1 | P2 | P3 | P4 | P5 | P6 | P7 | P8 | P9 | P10 | P11 | P12 |
| --- | --- | --- | --- | --- | --- | --- | --- | --- | --- | --- | --- | --- | --- | --- | --- | --- | --- | --- | --- | --- | --- | --- | --- | --- | --- | --- | --- |
| 121 | IgGMM | 60 | M | L | 2 | U | P | P | 69 | 37 | 41.5 | 18 | 9.3 | 25 |  | 10.43 | 0.60 | 9.08 | 5.45 | 36.20 | 22.51 | 1.41 | 5.28 | 5.85 | 0.76 | 1.27 | 1.16 |
| 122 | IgGMM | 57 | M | L | 3 | U | P | P | 68 | 60 | 106 | 25.8 | 3.2 | 53 |  | 17.40 | 0.04 | 30.78 | 11.17 | 12.51 | 23.18 | 0.32 | 1.32 | 2.39 | 0.17 | 0.25 | 0.46 |
| 123 | IgGMM | 60 | M | L | 2 | U | P | P | 82 | 348 | 81 | 26 | 2.8 | 65 |  | 1.74 | 0.09 | 20.76 | 3.16 | 13.70 | 53.82 | 1.35 | 3.55 | 0.77 | 0.07 | 0.81 | 0.17 |
| 124 | IgGMM | 75 | F | L | 3 | U | P | P | 99 | 50 | 70.8 | 28.5 | 6 | 63 |  | 6.38 | 0.47 | 23.45 | 6.85 | 21.75 | 33.15 | 2.11 | 3.02 | 1.54 | 0.13 | 0.79 | 0.35 |
| 125 | IgGMM | 80 | F | L | 3 | C | P | P | 75 | 20 | 81.5 | 39.3 | 4.4 | 84 |  | 2.06 | 0.20 | 3.56 | 2.20 | 35.72 | 42.15 | 1.94 | 7.25 | 2.04 | 0.24 | 2.05 | 0.60 |
| 126 | IgGMM | 67 | M | L | 2 | C | P | P | 131 | 114 | 60.7 | 41.6 | 8.1 | 53 |  | 4.03 | 0.17 | 7.30 | 15.34 | 28.35 | 31.60 | 1.09 | 6.22 | 3.19 | 0.27 | 1.67 | 0.77 |
| 127 | IgAMM | 68 | M | K | 2 | C | P | P | 82 | 285 | 62.5 | 20.9 | 3.4 | 40 |  | 2.13 | 0.17 | 1.61 | 2.74 | 31.50 | 12.43 | 30.65 | 6.17 | 7.74 | 0.53 | 1.98 | 2.36 |
| 128 | IgAMM | 54 | F | K | 2 | U | P | P | 57 | 174 | 97.6 | 27.2 | 6.8 | 59 |  | 0.82 | 0.14 | 0.18 | 1.16 | 15.77 | 25.39 | 53.89 | 1.55 | 0.41 | 0.10 | 0.43 | 0.16 |
| 129 | IgAMM | 73 | M | K | 2 | U | N | P | 48 | 21 | 72.6 | 46.5 | 5.5 | 54 |  | 11.98 | 1.27 | 4.08 | 3.95 | 38.72 | 13.52 | 12.89 | 6.45 | 3.90 | 0.39 | 1.83 | 1.04 |
| 130 | IgAMM | 67 | F | K | 2 | C | P | P | 122 | 164 | 73.5 | 38.2 | 3.9 | 53 |  | 3.31 | 0.50 | 1.55 | 2.70 | 40.90 | 22.17 | 17.78 | 5.12 | 3.37 | 0.50 | 1.25 | 0.85 |
| 131 | IgAMM | 52 | M | L | 2 | C | P | P | 142 | 152 | 75.4 | 34 | 3.2 | 77 |  | 5.12 | 0.14 | 3.89 | 3.79 | 34.76 | 22.89 | 18.75 | 3.94 | 4.54 | 0.25 | 1.00 | 0.92 |
| 132 | IgAMM | 62 | M | K | 2 | C | P | P | 97 | 153 | 89.2 | 34.9 | 6.5 | 82 |  | 4.03 | 0.91 | 1.89 | 2.70 | 39.71 | 23.87 | 18.23 | 5.70 | 0.94 | 0.14 | 1.70 | 0.16 |
| 133 | IgAMM | 62 | M | K | 2 | U | P | P | 97 | 209 | 84.5 | 33.3 | 3.5 | 81 |  | 2.21 | 0.34 | 0.62 | 2.20 | 45.66 | 33.35 | 7.44 | 5.88 | 0.81 | 0.07 | 1.37 | 0.05 |
| 134 | IgAMM | 54 | F | L | 1 | C | P | P | 88 | 52 | 83.1 | 36.9 | 7.6 | 106 |  | 4.58 | 0.35 | 8.41 | 3.87 | 42.52 | 21.33 | 1.63 | 9.12 | 4.74 | 0.51 | 2.04 | 0.90 |
| 135 | IgAMM | 70 | M | K | 2 | C | N | N | 132 | 91 | 59.9 | 34.1 | 2.1 | 55 |  | 8.39 | 0.98 | 4.93 | 3.76 | 36.52 | 21.21 | 10.97 | 4.22 | 5.45 | 1.25 | 1.03 | 1.28 |
| 136 | IgAMM | 66 | M | K | 1 | C | N | P | 146 | 150 | 57.1 | 38.5 | 4.7 | 76 |  | 4.08 | 0.60 | 2.14 | 3.15 | 49.68 | 15.15 | 5.33 | 11.74 | 4.81 | 0.46 | 2.02 | 0.84 |
| 137 | IgAMM | 72 | M | K | 1 | C | N | P | 137 | 173 | 68.7 | 43.3 | 3.5 | 55 |  | 13.53 | 1.33 | 4.49 | 4.34 | 40.99 | 18.47 | 5.20 | 6.63 | 2.28 | 0.34 | 1.77 | 0.64 |
| 138 | IgAMM | 66 | M | K | 1 | C | N | N | 123 | 143 | 63.9 | 41.4 | 13.6 | 77 |  | 4.14 | 0.70 | 3.66 | 4.47 | 44.90 | 23.97 | 4.72 | 8.44 | 2.36 | 0.35 | 1.87 | 0.42 |
| 139 | IgAMM | 75 | F | L | 3 | C | N | N | 74 | 131 | 50.8 | 26.3 | 11.7 | 237 |  | 14.79 | 1.75 | 5.70 | 4.05 | 39.13 | 14.63 | 4.42 | 5.07 | 6.40 | 0.42 | 1.74 | 1.90 |
| 140 | IgAMM | 81 | M | K | 3 | C | P | P | 78 | 79 | 76.8 | 29.8 | 9.3 | 114 |  | 4.09 | 0.79 | 1.24 | 2.29 | 34.86 | 13.45 | 33.58 | 3.14 | 4.36 | 0.21 | 0.98 | 1.01 |
| 141 | IgAMM | 65 | F | L | 1 | C | N | P | 134 | 134 | 70.1 | 44.8 | 3.3 | 45 |  | 8.10 | 1.09 | 2.64 | 2.80 | 49.38 | 18.23 | 5.35 | 8.05 | 1.35 | 0.20 | 2.41 | 0.40 |
| 142 | IgAMM | 74 | F | K | 2 | C | P | P | 87 | 173 | 89.5 | 27.1 | 2.2 | 40 |  | 1.06 | 0.38 | 0.37 | 3.85 | 30.55 | 45.08 | 12.87 | 2.68 | 1.85 | 0.16 | 0.65 | 0.49 |
| 143 | IgAMM | 65 | F | K | 1 | C | N | P | 112 | 221 | 60.8 | 40.4 | 4.3 | 20 |  | 7.97 | 1.14 | 5.10 | 5.94 | 42.40 | 20.93 | 3.19 | 8.31 | 1.64 | 0.37 | 2.53 | 0.49 |
| 144 | IgAMM | 68 | F | L | 3 | C | P | P | 112 | 190 | 86.1 | 27.1 | 4.2 | 58 |  | 3.44 | 0.37 | 1.42 | 4.17 | 46.35 | 28.35 | 8.34 | 3.95 | 1.88 | 0.27 | 1.00 | 0.46 |
| 145 | IgAMM | 88 | F | K | 3 | C | P | P | 67 | 44 | 73.7 | 38.8 | 11.3 | 172 |  | 3.99 | 0.69 | 2.15 | 3.01 | 45.69 | 23.15 | 8.94 | 7.31 | 2.49 | 0.25 | 1.71 | 0.62 |
| 146 | IgAMM | 78 | M | L | 3 | U | N | P | 84 | 65 | 54.4 | 32.7 | 17.5 | 451 |  | 4.45 | 0.75 | 2.19 | 3.69 | 52.22 | 14.85 | 3.47 | 5.60 | 9.33 | 0.36 | 1.28 | 1.81 |
| 147 | IgAMM | 78 | M | L | 3 | U | P | P | 68 | 82 | 74.9 | 26 | 4.2 | 78 |  | 0.75 | 0.15 | 0.56 | 1.88 | 38.82 | 38.38 | 10.01 | 2.15 | 6.37 | 0.18 | 0.56 | 0.19 |
| 148 | IgAMM | 86 | M | L | 2 | C | P | P | 94 | 160 | 37.3 | 16 | 5.9 | 69 |  | 1.49 | 0.18 | 0.80 | 2.17 | 48.75 | 21.29 | 11.81 | 5.83 | 5.55 | 0.36 | 0.98 | 0.78 |
| 149 | IgAMM | 42 | F | L | 2 | C | N | N | 107 | 156 | 53.8 | 28.5 | 4.5 | 42 |  | 2.72 | 0.25 | 1.22 | 4.09 | 51.03 | 15.55 | 3.82 | 13.69 | 2.66 | 0.28 | 3.98 | 0.70 |
| 150 | IgAMM | 83 | M | K | 3 | C | N | P | 90 | 142 | 46.6 | 18.8 |  |  |  | 6.33 | 0.58 | 2.29 | 3.98 | 44.10 | 23.92 | 6.07 | 7.16 | 3.10 | 0.30 | 1.57 | 0.61 |

| NO | group | age | gender | LC | ISS stage | chemo | SPE | IFE | Hb | PLT | TP | ALB | Urea | CREA | SA | P1 | P2 | P3 | P4 | P5 | P6 | P7 | P8 | P9 | P10 | P11 | P12 |
| --- | --- | --- | --- | --- | --- | --- | --- | --- | --- | --- | --- | --- | --- | --- | --- | --- | --- | --- | --- | --- | --- | --- | --- | --- | --- | --- | --- |
| 151 | IgAMM | 58 | F | L | 2 | U | P | P | 73 | 211 | 96.4 | 30.4 | 3.1 | 43 |  | 6.92 | 0.97 | 4.18 | 4.36 | 45.17 | 15.82 | 2.42 | 14.29 | 1.37 | 0.35 | 3.83 | 0.32 |
| 152 | IgAMM | 54 | M | L | 2 | C | N | P | 147 | 237 | 76.5 | 41.2 | 8.9 | 102 |  | 7.52 | 1.20 | 4.34 | 5.02 | 40.73 | 19.35 | 7.04 | 6.08 | 5.26 | 0.56 | 1.76 | 1.17 |
| 153 | IgAMM | 62 | F | K | 2 | U | P | P | 104 | 160 | 88.7 | 24.3 | 8.2 | 35 |  | 2.86 | 0.17 | 0.87 | 3.42 | 44.21 | 31.01 | 11.40 | 3.61 | 0.72 | 0.39 | 0.97 | 0.37 |
| 154 | IgAMM | 47 | M | K | 1 | C | N | P | 127 | 145 | 59.5 | 38.6 | 5.5 | 59 |  | 7.23 | 1.10 | 5.39 | 4.04 | 43.77 | 19.11 | 6.29 | 4.65 | 5.55 | 0.86 | 0.98 | 1.02 |
| 155 | IgAMM | 55 | M | L | 3 | C | P | P | 117 | 99 | 106.1 | 22 | 3.9 | 95 |  | 0.55 | 0.12 | 0.12 | 1.06 | 16.83 | 29.57 | 48.33 | 1.61 | 1.25 | 0.08 | 0.42 | 0.08 |
| 156 | IgAMM | 67 | M | K | 2 | C | N | P | 103 | 45 | 57 | 34 | 3.2 | 81 |  | 2.83 | 0.74 | 1.20 | 3.11 | 50.87 | 14.68 | 8.23 | 7.40 | 6.91 | 0.57 | 1.80 | 1.66 |
| 157 | IgAMM | 71 | F | L | 1 | U | P | P | 133 | 793 | 83.4 | 38.8 | 4.6 | 64 |  | 5.20 | 0.71 | 3.86 | 3.97 | 37.22 | 22.83 | 15.25 | 7.23 | 1.24 | 0.24 | 1.88 | 0.37 |
| 158 | IgAMM | 67 | M | K | 3 | U | P | P | 63 | 99 | 79.3 | 28.6 | 5.9 | 152 |  | 1.54 | 0.21 | 0.34 | 2.13 | 29.21 | 38.82 | 22.46 | 3.39 | 0.64 | 0.13 | 0.88 | 0.26 |
| 159 | IgAMM | 82 | F | K | 3 | C | P | P | 79 | 92 | 90.7 | 25 | 4 | 79 |  | 6.39 | 0.95 | 1.49 | 1.79 | 19.29 | 12.12 | 51.56 | 2.75 | 1.97 | 0.48 | 0.72 | 0.49 |
| 160 | IgAMM | 81 | F | L | 3 | C | N | P | 117 | 401 | 59.6 | 25.5 | 6.5 | 95 |  | 3.65 | 0.22 | 1.77 | 2.92 | 47.47 | 19.78 | 7.57 | 7.60 | 4.81 | 0.44 | 2.35 | 1.42 |
| 161 | IgAMM | 71 | M | K | 1 | C | P | P | 151 | 158 | 80.2 | 42.5 | 5.8 | 62 |  | 2.03 | 0.51 | 2.86 | 2.84 | 32.02 | 35.79 | 15.52 | 4.24 | 2.53 | 0.18 | 1.00 | 0.49 |
| 162 | IgAMM | 61 | M | L | 1 | U | N | P | 36 | 268 | 61.7 | 35.6 | 5.2 | 39 |  | 5.16 | 0.76 | 4.49 | 3.49 | 39.89 | 22.41 | 9.94 | 5.88 | 4.25 | 0.53 | 1.96 | 1.24 |
| 163 | IgAMM | 57 | F | L | 1 | U | P | P | 102 | 202 | 87.9 | 42 | 4 | 44 |  | 6.73 | 1.14 | 2.72 | 3.55 | 44.15 | 23.17 | 8.48 | 6.34 | 1.33 | 0.19 | 1.96 | 0.25 |
| 164 | IgAMM | 72 | M | L | 1 | C | N | N | 91 | 68 | 63.7 | 36 | 7.6 | 60 |  | 4.94 | 0.52 | 1.73 | 2.92 | 49.34 | 18.11 | 3.90 | 11.44 | 2.24 | 0.34 | 3.83 | 0.67 |
| 165 | IgAMM | 78 | F | L | 2 | C | P | P | 78 | 148 | 65.9 | 28.9 | 7.1 | 72 |  | 4.45 | 1.02 | 1.88 | 2.89 | 44.33 | 19.10 | 9.00 | 6.92 | 6.37 | 0.38 | 2.09 | 1.56 |
| 166 | IgAMM | 61 | F | L | 3 | U | P | P | 81 | 144 | 107 | 24.1 | 10.8 | 87 |  | 3.04 | 0.38 | 0.68 | 2.05 | 51.22 | 31.19 | 1.60 | 5.23 | 1.01 | 0.49 | 1.37 | 1.73 |
| 167 | IgAMM | 88 | M | K | 3 | C | P | P | 70 | 62 | 67.5 | 28.2 | 7.7 | 116 |  | 3.00 | 0.38 | 1.45 | 2.66 | 43.76 | 16.09 | 16.00 | 5.64 | 6.05 | 0.33 | 2.39 | 2.26 |

Note: Abbreviations: LC, light chain; ISS, International Staging System; chemo, chemotherapy; SPE, serum protein electrophoresis; IFE, immunofixation electrophoresis;

Hb, hemoglobin (g/L); PLT, platelet (×10^9^/L); TP, total protein (g/L); ALB, albumin (g/L); BUN, blood urea nitrogen(mmol/L);

Crea, Creatinine (μmol/L); SA, sialic acid (mg/dl); P1-P12, peak1-peak12.

a: group: HC, healthy control; LCMM, light chain multiple myeloma; IgGMM, IgG type multiple myeloma; IgAMM, IgA type multiple myeloma

b: gender: F, Female; M, Male;

c: LC: K, kappa light chain; L, lambda light chain

d: chemo: U: untreated; C, chemotherapy treated

e: SPE/IFE: P, positive; N, negative
